# Explainable machine learning analysis reveals sex and gender differences in the phenotypic and neurobiological markers of Cannabis Use Disorder

**DOI:** 10.1038/s41598-022-19804-2

**Published:** 2022-09-17

**Authors:** Gregory R. Niklason, Eric Rawls, Sisi Ma, Erich Kummerfeld, Andrea M. Maxwell, Leyla R. Brucar, Gunner Drossel, Anna Zilverstand

**Affiliations:** 1 Department of Psychiatry and Behavioral Sciences, University of Minnesota, 717 Delaware St. SE, Minneapolis, MN 55414 USA; 2 Institute for Health Informatics, University of Minnesota, Minneapolis, MN USA; 3 Medical Scientist Training Program, University of Minnesota, Minneapolis, MN USA; 4 Graduate Program in Neuroscience, University of Minnesota, Minneapolis, MN USA; 5 Medical Discovery Team on Addiction, University of Minnesota, Minneapolis, MN USA

**Keywords:** Biomarkers, Psychiatric disorders, Addiction

## Abstract

Cannabis Use Disorder (CUD) has been linked to a complex set of neuro-behavioral risk factors. While many studies have revealed sex and gender differences, the relative importance of these risk factors by sex and gender has not been described. We used an “explainable” machine learning approach that combined decision trees [gradient tree boosting, XGBoost] with factor ranking tools [SHapley’s Additive exPlanations (SHAP)] to investigate sex and gender differences in CUD. We confirmed that previously identified environmental, personality, mental health, neurocognitive, and brain factors highly contributed to the classification of cannabis use levels and diagnostic status. Risk factors with larger effect sizes in men included personality (high openness), mental health (high externalizing, high childhood conduct disorder, high fear somaticism), neurocognitive (impulsive delay discounting, slow working memory performance) and brain (low hippocampal volume) factors. Conversely, risk factors with larger effect sizes in women included environmental (low education level, low instrumental support) factors. In summary, environmental factors contributed more strongly to CUD in women, whereas individual factors had a larger importance in men.

## Introduction

Cannabis is the most commonly used illicit drug in the United States, with an estimated 8.2% of the population reporting cannabis use in the past month^1^. Of those who endorsed past-year use, an estimated 30.6% met criteria for Cannabis Use Disorder (CUD)^2^. Although men have historically reported a greater prevalence of cannabis use relative to women, this gender gap is narrowing^3–6^. Moreover, research shows that women progress to CUD more quickly than men^7,8^. While the observed broad differences in use patterns thus suggest that different factors may be underlying CUD in women versus men, little is known about which factors drive these differences^9,10^. Here we examine both neurobiological (e.g., sex-specific) risk factors as well as environmental (e.g., gender-specific) risk factors that are difficult to parse in humans^11^. We will refer to the differences between men and women in this study as sex/gender differences to more accurately reflect this complexity.

The factors underlying cannabis use and dependence are complex and likely include neurobiological, individual-level (e.g., personality, cognitive), and environmental risk factors. These factors have, until now, often only been investigated in a fragmented way, with researchers focusing on a small number of factors in each study or focusing on a single domain of interest. However, the recent availability of large public datasets with broad phenotyping, such as the Human Connectome Project (HCP), and the emergence of machine learning approaches and ranking tools for evaluating the importance of a large number of factors and their relative contributions^12^, make it possible to shift toward an analysis of the patterns of factors underlying CUD.

Prominent neurobiological theories of addiction have traditionally focused on the importance of reward- and approach-related behavior, with newer theories integrating cognitive and affective factors as important additional functional domains^13–16^. However, even “multi-mechanistic” addiction models are limited, such as the Koob–Volkow model^13^ that discusses three main mechanisms involved in addiction: incentive salience/habit formation (reward/approach-related behavior), negative affect, and executive function. Only very few addiction theories have moved beyond this triadic-mechanism framework [e.g. see “vulnerabilities in decision making”^17^ as an example], and even less empirical work has been done using a multi-domain data-driven approach [e.g., see “An Integrated Multimodal Model of Alcohol Use Disorder”^18^ as an example]. However, separate empirical investigations strongly suggest the involvement of a myriad of different factors.

Individual risk factors that have been shown to predict high likelihood of cannabis abuse and dependence include sex/gender^19^, general cognitive ability [IQ/working memory^20–22^], childhood mental health disorders [(depression, externalizing/conduct disorder)^19,23–28^], trauma history^26,29,30^ and stressful life events/low socioeconomic status^23^. Cannabis users have further been characterized to have personality traits of high openness/extraversion and low agreeableness/conscientiousness, while neuroticism has not been linked to cannabis abuse^31–33^. Increased openness in particular appears to discriminate cannabis users from other drug users^33^. The triadic neurobiological models of cannabis addiction are supported by evidence on increased reward/approach-related behavior [e.g., increased sensation seeking^34^ & delay discounting^35,36^], a role of increased negative affect [e.g. increased prevalence of depression^2,19,23^] and deficits in executive function, specifically deficits in memory/working memory performance and processing speed deficits that predict risk for chronic cannabis use^20–22,37,38^. Neuroimaging studies corroborate these theories by demonstrating an upregulation of brain regions involved in reward/approach-related behavior [e.g., salience/reward network^16,39^] and structural changes in valuation networks [e.g., orbitofrontal cortex^40,41^], as well as changes in brain structures supporting memory function [e.g., reduced hippocampal volume^40^; altered memory network function^39^]. Finally, reduced educational attainment and lower socioeconomic status have been shown to co-occur with chronic cannabis use^42–44^. Specifically, longitudinal studies have concluded that common risk factors [e.g., lack of support in family/peer/school environment^45^ and mental health issues^43^] cause both substance use and lower educational attainment/socioeconomic status.

Although sex/gender differences in use patterns in CUD are increasingly well established, it is unclear which neurobiological, individual-level (e.g., personality, cognitive) and environmental mechanisms drive these differences. Preclinical models of cannabis use and dependence suggest that neurobiological factors may contribute to these sex/gender differences. Female rodents metabolize ∆9-tetrahydrocannabinol (THC) at a faster rate than males^46,47^. Behaviorally, female rodents also demonstrate more cannabis withdrawal symptoms^48^, higher rates of cannabinoid reinstatement after abstinence^49^, and higher rates of self-administration relative to males^50^. Further, while sex/gender differences have not been studied comprehensively in adults to date^9^, a machine-learning analysis of the predictors of initiation of cannabis use in adolescence found distinct neurobiological, individual-level and environmental risk factor profiles in boys versus girls^10^. Specifically, individual level factors such as sensation/novelty seeking were predictive of cannabis use onset in boys, whereas factors that are more closely linked to the environment, such as verbal IQ, sexual relationships and parent personality, were predictive in girls^10^. Finally, gendered environmental experiences may also influence CUD. For example, women with substance use disorders are more likely to experience a lack of social support and increased isolation relative to men^9,11^. The endocannabinoid system is essential in regulating stress^51,52^. Stressful environmental factors (e.g., lack of social support) may contribute to cannabis use and dependence in a sex/gender-specific way through altered endocannabinoid signaling. Therefore, in this study, we comprehensively examine sex/gender differences in the relative contribution of neurobiological, individual, and environmental risk factors to high cannabis use levels and CUD to fill this gap in the literature.

To evaluate the relative importance of a wide variety of factors associated with high cannabis use levels and cannabis dependence as well as potential sex/gender differences in a well-described community sample (HCP^39^; N = 1204), we employed state-of-the-art machine learning methods [XGBoost (eXtreme Gradient Boosting)^53^, a tree-based ensemble machine learning algorithm] in combination with a ranking tool [SHapley’s Additive exPlanations (SHAP)^12^] to assign relative importance (i.e., SHAP values) to each of the associated factors. Decision and boosted tree-based machine learning methods are powerful tools for identifying associated factors in psychiatric research due to their non-parametric nature (resilience to non-normal data distributions) and their tolerance for multicollinear and missing data^54^. However, when used on their own, it is difficult to interpret the relative importance of each of the factors involved. We therefore employed SHAP, an extension of methodology originally developed for consistent credit attribution in cooperative game theory^55^, to provide a reliable and consistent ranking of the unique relative importance of each factor^12^. In addition to providing a ranking for the unique and additive importance of all identified factors, SHAP allows for examining interactions between factors in a model^56^, such as sex/gender-related interactions. In summary, the current study is an exploratory, data-driven analysis that leverages state-of-the-art machine learning algorithms to model the complex factors underlying chronic cannabis use and their relative importance by sex/gender.

## Methods

### Participants

We analyzed data from the final HCP data release [N = 1204, aged 22–35, 54% female; https://db.humanconnectome.org/data/projects/HCP_1200; HCP preprocessing pipeline (4.1)]. The data was collected in 2012–2015 at Washington University in St. Louis, Missouri, United States. All subject recruitment procedures and informed consent forms, including consent to share de-identified data, were approved by the Washington University Institutional Review Board (IRB) in accordance with the Declaration of Helsinki. For the present study, after permission was obtained from the HCP to use the Open Access and Restricted Access data for the present study (see Data Availability Statement below), a protocol filed with the University of Minnesota Institutional Review Board (IRB) met criteria for exemption. In this community sample, 9% of participants met the DSM-IV criteria for cannabis dependence (n = 109, 26% female; note that cannabis abuse was not assessed). The HCP study sample had a similar racial and socioeconomic status distribution as reported in the 2010 United States Census (Census United States 2010: 72% White, 13% Black/African–American, 6% Asian/Nat. Hawaiian/ Other Pacific Islander, 9% other; median income (25–34 year olds) = $49,445; mean education years (25–34 year olds) = 13.8 years). See Table 1 for detailed demographic information. See Supplementary Fig. 1 for an overview of the analysis flow.

**Table 1:** Demographic characteristics of the full sample who completed the SSAGA interview (n = 1204).

	Full sample	CUD (n = 109)	Control (n = 1095)	CUD − control difference
Sex/gender (M/W)	549/655	81/28	468/627	χ^2^ = 39.84, *p* < .001*
Race (White, Black/African–American, Asian/Nat. Hawaiian/ Other Pacific Islander, Other)	885/193/69/57	83/15/1/10	802/178/68/47	χ^2^ = 11.14, *p* = .053
Age of first cannabis use (< = 14, 15–17, 18–20, > = 21, never)	81/240/207/126/550	35/55/16/3/0	46/185/191/123/550	χ^2^ = 232.80, *p* < .001*
THC urine status (positive/negative)	1062/142	66/43	996/99	χ^2^ = 88.11, *p* < .001*
Lifetime cannabis use (0, 1–9, 10–99, 100–999, 1000 +)	550/324/144/78/108	0/0/19/29/61	550/324/125/49/47	χ^2^ = 460.01, *p* < .001*
Age (mean ± SD)	28.84 ± 3.69	28.58 ± 3.53	28.86 ± 3.71	*t*(1202) = 0.76, *p* = .45
Education years (mean ± SD)	14.86 ± 1.82	14.22 ± 4.39	14.93 ± 1.80	*t*(1202) = 3.90, *p* < .001*
Income (mean ± SD)^a^	5.00 ± 2.17	4.39 ± 2.37	5.06 ± 2.14	*t*(1197) = 3.08, *p* = .002*

	Full sample	CUD (n = 109)	Control (n = 1095)	Men − women difference
Men: age of first cannabis use (≤ 14, 15–17, 18–20, ≥ 21, never)	49/127/96/59/218	25/43/11/2/0	24/84/85/57/218	χ^2^ = 5.88, *p* = .12
Women: age of first cannabis use (≤ 14, 15–17, 18–20, ≥ 21, never)	32/113/111/67/332	10/12/5/1/0	22/101/106/66/332	χ^2^ = 5.88, *p* = .12
Men: lifetime cannabis use (0, 1–9, 10–99, 100–999, 1000 +)	218/130/78/46/77	0/0/12/23/46	218/130/66/23/31	χ^2^ = 50.67, *p* < .001*
Women: lifetime cannabis use (0, 1–9, 10–99, 100–999, 1000 +)	332/194/66/32/31	0/0/7/6/15	332/194/59/26/16	χ^2^ = 50.67, *p* < .001*

*Significant CUD-Control Difference or Men-Women Difference.

^a^Income was binned (1 =  < 10 k, 2 = 1.1–19.999 k, 3 = 20–29.999 k, 4 = 30–39.999 k, 5 = 40–49.999 k, 6 = 50–74.999 k, 7 = 75–99.999 k, 8 =  ≥ 100 k). Five control participants did not report income.

### Outcome variables

Our primary outcome measures of interest were (1) lifetime level of cannabis use and (2) lifetime diagnosis of cannabis dependence, which were assessed using a structured interview (the Semi-Structured Assessment for the Genetics of Alcoholism [SSAGA]^57^). Level of cannabis use was assessed by the reported number of lifetime uses (categories: 0, 1–5, 6–10, 11–100, 101–999, 1000 + lifetime uses). For our analysis, we merged two categories, such that we had five different levels of cannabis use on a logarithmic scale (0, 1 + , 10 + . 100 + , 1000 + lifetime uses). Classification analyses were conducted for each outcome respectively, i.e., we classified escalation of cannabis use and dependence (1 + uses, 10 + uses, 100 + uses, 1000 + uses, and DSM-IV dependence). Each analysis classified a binary outcome using the entire sample; that is, we classified individuals who used cannabis 1 + times from those who did not, classified individuals who used cannabis 10 + times from those who used cannabis < 10 times, and so on. The smallest number of cases for considering sex/gender interaction effects were hence found in the 1000 + model (cases: N = 31 women, N = 77 men), and in the model with cannabis dependence diagnosis as an outcome (cases: N = 28 women, N = 81 men).

### Phenotypic models

The HCP dataset contains a wide array of self-report, diagnostic and behavioral measures assessing domains of cognition, emotion, social function, psychiatric dysfunction, and personality^58^. To examine as broad a phenotypic space as possible, this study used all available behavioral, self-report, and interview-based measures in the HCP database (including all in-scanner task behavior variables). We generally included both summary scores and subscale/item-level scores, because the machine learning method we used (detailed below) explicitly allows for correlated factors during model fitting^53^. This allows for a direct comparison of the contribution of summary scores versus subscale/item-level scores to the classification. For a complete list of all included phenotypic variables (273 in total), see Supplementary Table 1.

### Freesurfer (structural MRI) models

For our structural Magnetic Resonance Imaging (MRI) or “Freesurfer” model, we used the Freesurfer data provided by HCP^59,60^. These summary data included Freesurfer-generated volume estimates for 44 regions and surface area and cortical thickness estimates for 68 regions. We did not correct these measures for total brain volume, to avoid introducing artificial sex/gender differences due to overcorrection^61^, but did include 19 summary measures including total gray matter volume, white matter volume, and brain segmentation volume as additional factors in the model (199 factors total).

### Resting-state global and local efficiency models

We used the resting-state functional MRI (rsfMRI) data as preprocessed by HCP^59^ in the volumetric data format. Using the Brain Connectivity Toolbox^48^, we conducted a graph theory analysis to extract measures of nodal global and local efficiency [connectivity of a brain region with the rest of the network (global) or with the network within a small neighborhood (local)] from 638 similarly sized brain regions [whole-brain, excluding cerebellum;^62,63^ sub-parcellation of the Automated Anatomical Labeling atlas (AAL)^64^]. For each participant, a 638-by-638 matrix of Fisher’s z-transformed Pearson correlations was computed, representing the normalized bivariate correlation of each brain region with each other region. This correlation matrix was binarized at a proportional cost (to improve stability of measures over absolute thresholds)^65^ of 0.15 (which is in the middle of the optimal range of 0.01–0.30)^66^, to represent the strongest 15% of positive connections. We characterized the intrinsic properties of the obtained connectivity graphs by computing nodal global and local efficiency for all 638 brain regions^67^, and then averaging both graph theory measures within each larger AAL region (90 factors).

### Resting-state network connectivity models

We used the resting-state grayordinate (CIFTI) functional data provided by HCP, to compute within and between functional connectivity for a set of brain networks^68–71^. We first parcellated the whole brain into 718 parcels using the Cole-Anticevic parcellation^72,73^. We calculated the pairwise Pearson correlations between each pair of parcels in the brain, normalized the obtained correlations using Fisher’s z-transform, and averaged the parcel-to-parcel correlation values both within and between networks (78 factors in total).

### Task fMRI models

All task fMRI (tfMRI) data were preprocessed by HCP using the same steps as for the rsfMRI data^59^. We used the provided task fMRI task activation Contrast Of Parameter Estimates (COPE) maps (generated by FSL’s FEAT) that were acquired during seven behavioral tasks, described in^58^. These tasks included (1) an N-Back task, (2) a gambling task, (3) a motor mapping task, (4) a language-math task, (5) a social cognition task, (6) a relational-processing task, and (7) an emotion-processing task. We selected 12 COPE maps that represented the main task effects of interest for each task: (1) N-Back task: 2back-0back contrast, (2) gambling task: response to punishments and rewards, (3) motor mapping task: response to left/right foot, left/right hand and tongue movements, (4) language-math task: story-math contrast, (5) social cognition task: social-random contrast, (6) relational-processing task: relational-match contrast, and (7) emotion-processing task: negative faces-shapes contrast. To define activation clusters, we employed the cifti-find-clusters command in Connectome Workbench v1.4.2 (https://www.humanconnectome.org/software/get-connectome-workbench) to find clusters of significantly activated voxels for each of the selected contrast maps, using the full sample (N = 889 with task fMRI). We chose a cutoff of Cohen’s d > 0.8 to select only clusters with large effect sizes and reduce the number of factors entering our final model. Then, for individual participants, we extracted the mean beta weight within each cluster of selected voxels. The task fMRI model contained 448 factors. For a complete list of all included fMRI task contrasts (12 in total), see Supplementary Table 2.

### Classification analysis using gradient tree boosting

To classify each outcome variable of interest, we used a nonparametric classification approach called gradient tree boosting. Gradient tree boosting machines are fit to the gradient of the loss function at every iteration, building up a series of simple models using gradient descent in function space. Specifically, we used the recently developed XGBoost^53^, a fast and scalable state-of-the-art gradient tree boosting system. We chose gradient tree boosting because this class of methods is stable and requires a much smaller sample size to produce reliable effect estimates^74^, compared to previous methods such as support vector machines (SVM)^75^. Specifically, simulations demonstrated that XGBoost was able to achieve a prediction accuracy of 0.90, detecting the top 27 relevant features out of ~ 2000 features reliably in a biological benchmarking dataset (N = 865) with as little as N = 20 for the training sample^74^. Comparative simulations using the identical benchmarking dataset further demonstrated a ~ 12-fold reduction in the needed training sample size with XGBoost as compared to SVM (e.g. with XGBoost N = 20 achieves > 0.90 accuracy, while N = 250 with SVM)^74,75^. Also, while class imbalance has been shown to lower the performance of XGBoost, comparative simulations demonstrated that performance was acceptable up to a 17:1 ratio for class imbalance in the population^76^. Another advantage of XGBoost is its ability to deal with the presence of missing values in the data through sparsity-aware split finding, capturing trends in missing values by the model^53^. It is therefore not necessary to use an imputation for missing values^53^. Finally, the feature ranking tool that we applied (see for details below) had been initially applied to XGBoost, which outperformed other machine learning models such as SVM, Lasso penalized linear logistic regression, or an unsupervised Parzen window method^77^.

Nested k-fold cross-validation was used to tune hyperparameters (inner loop) and evaluate classification performance (outer loop), generating an unbiased estimate of the model performance^78^. We used k = 5, therefore evaluating 5 models using an 80–20 train-test split in both inner and outer loops. Bentéjac and colleagues^79^ performed a comprehensive evaluation of parametrization tuning for XGBoost. They compared the XGBoost default parameter values with different tuning approaches and concluded that tuned models performed significantly better^79^. They then proposed new (optimized) default values for (a) learning rate [suggested values: 0.05, 0.1], (b) maximum tree depth [suggested value: 100 (unlimited)], and (c) subsampling [suggested value: 0.75]^79^. These proposed values are conservative (reducing the risk of model overfitting) for learning rate and subsampling, but non-conservative for maximum tree depth. We therefore chose to center our grid search around their proposed values for learning rate and subsampling, but used more conservative (smaller) values for maximum tree depth than proposed. Overall, we hence chose a conservative approach, aimed at preventing model overfitting. We considered the Cartesian product of the following hyperparameters: learning rate = {0.01, 0.02, 0.05, 0.1, 0.2}, max tree depth = {4, 6, 8, 10, 12}, and subsampling size = {0.6, 0.8, 1}. During the inner loop of the nested cross-validation, we conducted a grid search to determine the best combination of the above hyperparameters. The performance of the best model selected from the inner loop was evaluated in the outer loop, resulting in 5 performance estimates. The overall best performing set of hyperparameters for each outcome is reported in the Supplementary Table 3. We additionally used an early stopping parameter of 30 rounds, thus preventing overfitting when the model loss function fails to improve (therefore number of trees was not included in the hyperparameter grid). Since the HCP dataset contains many related participants, our cross-validation scheme always assigned family members to the same group (train or test) for every fold, therefore ensuring that test performance was not inflated by allowing the model to be trained and then tested on a related subject.

We quantified the performance of each model by using the Area Under the Curve of the Receiver Operating Characteristic Curve (AUC-ROCC), which describes how well the model can distinguish between classes. The AUC-ROCC ranges from 0 to 1; higher AUC-ROCCs indicate better predictive performance. An AUC-ROCC of 0.5 indicates random prediction for a binary outcome.

### Factor importance ranking using SHapley Additive exPlanations

Advanced machine learning methods such as gradient boosting machines are capable of making highly accurate predictions, but often these predictions come at the expense of interpretability. That is, traditional classification approaches do not allow for an interpretation of the relative importance of the factors involved, as they only evaluate the predictive performance of the entire model. To evaluate the unique relative importance of each model factor (referred to as “features” in machine learning research), we used SHAP (SHapley Additive exPlanations), proposed by^12^, as a feature ranking tool. SHAP provides an explanation model that computes the unique and additive importance of each model feature (predictive factor) in determining the final classification result. SHAP is based on the concept of Shapley Values, originally described in^55^ as a consistent method to allocate credit to a set of team members for a cooperative outcome. In this case, rather than the consortium consisting of a team of players working toward a common goal, the consortium consists of the set of features (factors) which work toward the common goal of producing the classification output of the model. The impact of each feature on the output of the model is defined as the change in model output when the feature is known, as opposed to unknown. Shapley values are the only currently available feature ranking tool that obeys a specific set of properties [local accuracy, consistency, and missingness^12^], which are considered desirable in explaining the output of a machine learning classification model. In combination with gradient-boosting machines such as XGBoost, this method is both robust to outliers and flexible^77^. An in-depth explanation of the properties of SHAP is beyond the scope of the current paper; for a full explanation of the properties of SHAP, the reasons these properties are desirable, and the equations used to derive the feature importance rankings, please see^12^ and^56^.

### Using SHAP to investigate sex/gender effects in cannabis use and dependence

Critical to our current investigation, SHAP is also able to leverage the assumption of feature additivity to compute interaction effects between sets of two factors in the model^56^. SHAP values can provide a rich alternative to traditional partial dependence plots^80^. While partial dependence plots only allow for an interpretation of how the output of a model depends on the interaction between two factors, SHAP dependence plots allow for interpreting interaction effects while accounting for both lower- and higher-order interaction effects of all factors in the model. In this study, we leveraged this to investigate sex/gender differences in model factors, as sex/gender was a strong predictor of cannabis outcomes in all models.

## Results

### Classification performance

**Figure 1:**
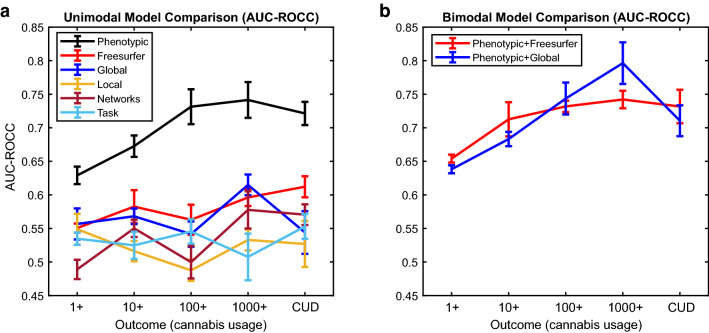
Area under the Curve of the Receiver Operating Characteristic Curve (AUC-ROCC) was used to quantify the classification performance of (**a**): Six unimodal models, and (**b**): Two additional multimodal models that combined the factors of the phenotypic model with the factors of the two best performing brain models. The phenotypic model performed better than any of the other unimodal models, and the inclusion of the two best sets of brain factors (Freesurfer & global efficiency) did not appreciably improve the performance of the phenotypic model. Error bars correspond to ± 1 Standard Error.

Cross-validated AUC-ROCCs of the six unimodal models we considered returned a wide range of performance indices (Fig. 1a). The phenotypic model had an average AUC-ROCC of 0.70 over all five outcome measures, and produced the best performance in classifying 1000 + cannabis uses (AUC-ROCC = 0.74). Of the brain models, the best performance was obtained by the Freesurfer (structural MRI) model (average AUC-ROCC = 0.58) and the global efficiency model (average AUC-ROCC = 0.57). The other brain models all performed similarly to each other, and were not considered further (AUC-ROCC range 0.52–0.53).

To determine if performance of the phenotypic model could be improved by adding factors from the most informative brain modalities, we then tested two bimodal models (phenotypic + Freesurfer, phenotypic + global efficiency; Fig. 1b). For both of the combined models, the average AUC-ROCC over all five outcomes was 0.71. The best performance of the combined models (phenotypic + Freesurfer, phenotypic + global efficiency) was obtained in classifying 1000 + cannabis uses (AUC-ROCC = 0.74 & 0.80, respectively). The results indicate that while the inclusion of brain data did not appreciably change the overall classification accuracy, specific brain factors (e.g. hippocampus volume, median rank = 4) were among the highest ranked predictors in these bimodal models.

### SHAP factor importance ranking

**Figure 2:**
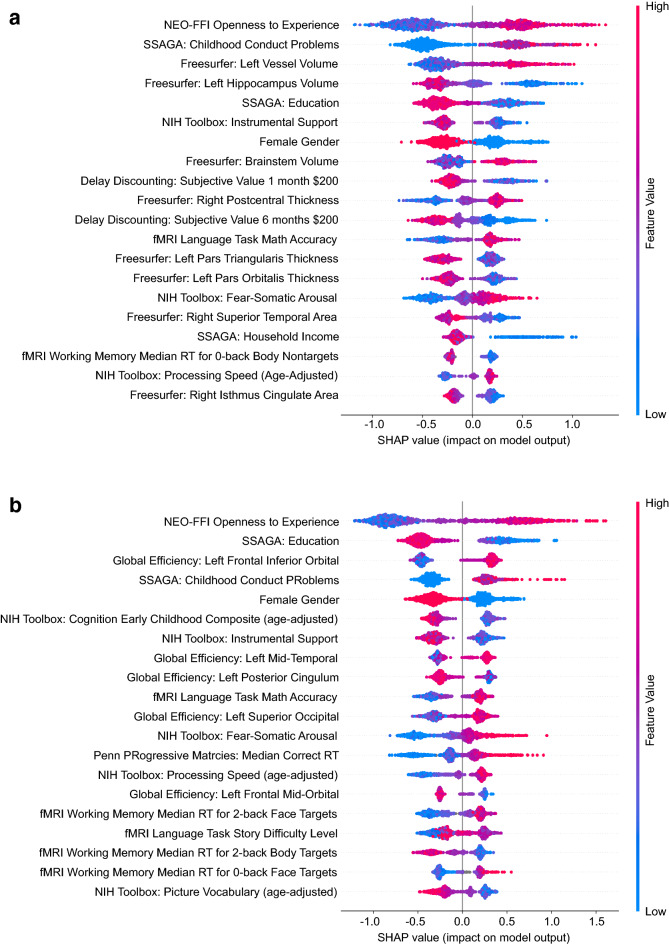
SHAP ranking for factors contributing to the two bimodal models (**a**: phenotypic + Freesurfer, **b**: phenotypic + global efficiency) classifying cannabis dependence. Higher positive SHAP values on the x-axis indicate that the observed factor pushed the classification closer towards cannabis dependence, whereas more negative SHAP values indicate that the factor pushed the classification away from cannabis dependence. Factors are ranked in order of the average (absolute) SHAP value, which indicates the importance of a factor. Individual data points (dots) represent the model output for each individual in the sample. The position of a dot on the x-axis represents the impact of the observed factor on the model output for the individual. The color of the individual dots represent the value of the observed measurements, with blue indicating lower and red higher values (e.g. red indicates high openness or high education level in these plots). As an example, in both plots high (red dots) observed “Openness to Experience” and low (blue dots) education levels pushed the model prediction closer towards cannabis dependence. That is, high “Openness to Experience” and lower education levels make it more likely that the model would categorize a given individual as cannabis-dependent relative to not dependent.

To determine which factors drove the performance of the best performing classification models, we used SHAP to estimate the relative importance of all factors (e.g., see the factors contributing to dependence in Fig. 2; see other models in Supplementary Figs. 2–9). To determine which factors consistently classified increased cannabis use levels and dependence, we computed the median rank of each factor across all models (see Supplementary Table 4). The consistent highly ranked factors across models (median rank ≤ 20, the default cutoff for highly ranked features in SHAP models) included a broad range of factors, such as sex/gender, environmental factors (income, education level), personality measures (openness), mental health measures (externalizing, childhood conduct disorder, aggression), neurocognitive measures (working memory, verbal IQ) and brain measures (hippocampal, brainstem and CSF volume; frontal pole thickness; insula, operculum and occipital resting-state connectivity) (Supplementary Table 4).

### SHAP sex/gender interaction analysis

Since sex/gender was a top ranked factor (ranked 4th across all phenotypic + Freesurfer and phenotypic + Global models, Supplementary Table 4), we examined interaction effects to identify sex/gender-specific factors that contribute to classifying cannabis dependence. We focused on the models predicting cannabis dependence and use levels of 1000 + lifetime uses as the most clinically relevant outcomes. We report all interaction effects with a SHAP interaction value of at least 0.1 (the sum of all SHAP values per model is 1), in order to discuss only interaction effects with meaningful effect sizes. When comparing effect sizes, we considered values <|0.1| as small, <|0.3| as moderate, <|0.5| as large and >|0.5| as very large effect sizes.

#### SHAP sex/gender interactions in models predicting cannabis dependence

**Figure 3:**
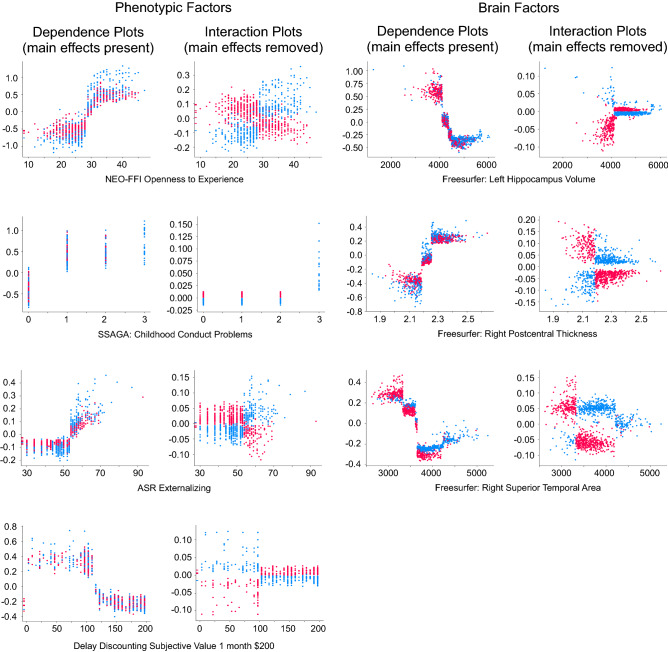
Several factors in the phenotypic + Freesurfer model classifying cannabis dependence showed sex/gender interaction effects. As in previous figures, each dot indicates an individual. Dots are colored according to sex/gender (red = women, blue = men). The left columns (“Dependence plots”) show individuals colored according to sex/gender, with main effects intact; the right columns (“Interaction plots”) again show the same individuals, but with main effects removed for improved visualization of the sex/gender interaction effects. The x-axis of the plots indicates the observed measurement value of each factor, and the y-axis of each plot indicates the SHAP value (where a higher SHAP value pushed the model closer towards dependence, and lower values pushed the model away from dependence). The magnitude of the SHAP values are standardized in the model and can therefore be interpreted as effect sizes.

**Figure 4:**
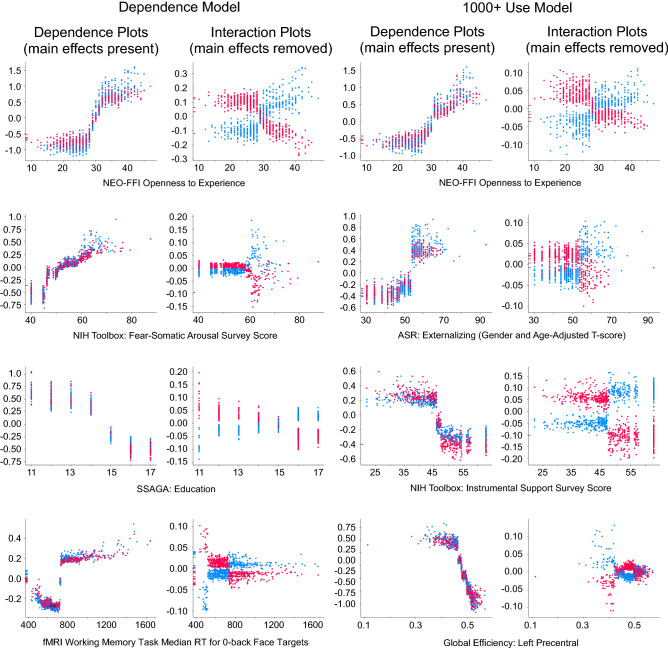
Several factors in the phenotypic + Global Efficiency model classifying cannabis dependence (left) and classifying heavy cannabis use (1000 + uses; right) showed sex/gender interaction effects. As in previous figures, each dot indicates an individual. Dots are colored according to sex/gender (red = women, blue = men). The left columns (“Dependence plots”) show individuals colored according to sex/gender, with main effects intact; the right columns (“Interaction plots”) again show the same individuals, but with main effects removed for improved visualization of sex/gender interaction effects. The x-axis of the plots indicates the observed measurement value of each factor, and the y-axis of each plot indicates the SHAP value (where a higher SHAP value pushed the model closer towards dependence, and lower values pushed the model away from dependence). The magnitude of the SHAP values are standardized in the model and can therefore be interpreted as effect sizes.

The bimodal models (phenotypic + Freesurfer; phenotypic + global) classifying cannabis dependence indicated sex/gender interaction effects for environmental factors (education level), personality measures (openness), mental health factors (childhood conduct disorder, fear somaticism), neurocognitive measures (delay discounting, working memory) and brain measures (hippocampal volume, postcentral thickness, superior temporal area) (Figs. 3, 4). Men as compared to women were more often classified as cannabis-dependent based on personality (high openness), mental health (high childhood conduct disorder, high fear somaticism), neurocognitive (impulsive delay discounting, slow working memory performance) and brain factors (low hippocampal volume, high postcentral thickness). In contrast, women were more often classified as dependent based on environmental (lower education level) and brain factors (smaller superior temporal area). Effect sizes for the main effects were often very large ( >|0.5|) for behavioral effects, and large ( >|0.3|) for brain effects (see Figs. 3, 4: *column “main effects present”*), while the sex/gender interaction effects had small to moderate effect sizes (Figs. 3, 4: *column “main effects removed”*). Overall, the direction of effects was therefore the same in men and women, but the sex/gender interaction effects indicated that the observed effects were much stronger in either men or women.

#### SHAP sex/gender interactions in models predicting heavy cannabis use

The 1000 + lifetime uses model demonstrated sex/gender interaction effects for environmental factors (instrumental support), personality measures (openness), mental health factors (externalizing) and brain measures (precentral efficiency) (see Fig. 4 for sex/gender interactions in the phenotypic + global model; the phenotyopic + Freesurfer model showed no sex/gender interaction effects > 0.1 ). Men as compared to women were more often classified as heavy cannabis users (+ 1000 uses) based on personality (high openness), mental health (high externalizing) and brain factors (low global efficiency of the precentral cortex). In contrast, women were more often classified as heavy cannabis users based on environmental factors (low instrumental support). Similarly as in the Cannabis Dependence models, the sex/gender interaction effects did not influence the directions of the effects but rather modulated the effect sizes such that the observed effects were much stronger in either men or women.

## Discussion

The current study used a machine learning approach to describe the complex factors underlying high cannabis use levels and dependence and their relative importance by sex/gender in a community sample of young adults in the United States. While a number of recent reviews have recognized the potential for machine learning methods in psychiatric research^81–86^, this is the first study to date to use such an approach in adults with CUD, although machine learning methods have been applied to examine adolescent cannabis use^10^. Therefore, it is also the first study to date to comprehensively study sex/gender differences in CUD in adults. Since conventional machine learning methods obtain increased predictive power at the cost of interpretability^77,83,87^, we paired our classification models [(XGBoost)^53^] with Shapley Additive eXplanations [(SHAP)^12^] to generate “explainable” machine learning models. This enables the ranking of factors (or “features”) according to their unique and additive importance in classifying an outcome.

Overall, the classification models achieved high accuracy, which in itself was remarkable since the used dataset was not designed to assess substance use and dependence [see Rawls and colleagues^18^ for a more in-depth discussion of the assessments and how they relate to addiction]. The current results further confirmed that a small number of factors, of the more than one thousand included in the analyses, consistently provided a unique and additive contribution to the classification performance, beyond other factors in the model. The identified factors included environmental, personality, mental health, neurocognitive and brain measures, demonstrating the complexity of the factors involved in CUD. Overall, the current results confirm the importance of multi-domain investigations into the factors underlying drug addiction, as in our previous empirical investigation of multi-domain factors in substance use disorders^18^.

Many factors that have been well described in the literature on CUD were replicated in this study, though we demonstrated here systematic sex/gender interaction effects for many factors for the first time. The environmental factors that most consistently contributed highly to model classification performance were income and education level. Previous longitudinal research further suggests that reduced educational attainment and lower socioeconomic status co-occur with (but do not directly cause) chronic cannabis abuse and dependence^42–45^. These current results further replicate previous work that has linked the personality trait openness to high cannabis use levels and dependence, suggesting that high openness is a predictor specifically for cannabis as a primary drug of choice^31–33^. Additionally, the current results also confirm an important role of externalizing mental health disorders, aggression, and a history of child conduct disorder, which have all been identified as risk factors for cannabis abuse and dependence in longitudinal research^24–28^. Notably, while our results provide additional support for an important role of externalizing disorders [e.g.^26–28^], we could not confirm a link between cannabis abuse or dependence and internalizing disorders, as had been reported by some other studies [e.g.^19,23^]. Further, in the current study, working memory and verbal IQ measures were among the most highly ranked neurocognitive factors, both of which have consistently been associated with CUD and shown to be risk factors for (not consequences of) cannabis abuse and dependence^20–22,37,38^. Finally, brain measures that were consistently highly ranked included hippocampal volume, an important structure of the brain’s memory system^39,88,89^, as well as brainstem volume, frontal pole thickness, insula, operculum and occipital resting-state connectivity, all of which are part of the reward, salience and visual brain networks that are most densely innervated by dopaminergic receptors^90^. These results converge with previous studies and systematic reviews that have demonstrated that CUD is characterized by changes in the brain’s memory system^39,40,91^, the reward and salience networks^39,41^, and the occipital lobe^92,93^. These results also demonstrate changes in the brain’s reward/approach-related system, a domain that was not captured well by the behavioral assessments or neuroimaging tasks used in this study [see Rawls and colleagues^18^ for a more in-depth discussion]. Thus, the current evidence supports the triadic models of cannabis addiction by indicating changes in the brain’s reward/approach system, deficits in executive function, specifically in working memory function and verbal IQ, and a role of negative affect, specifically of externalizing symptoms and aggression.

The analysis of sex/gender interaction effects revealed complex sex/gender differences in the multi-domain factors underlying cannabis abuse and dependence. Environmental factors such as educational attainment and instrumental support (the latter was not among the highest ranked factors overall) were factors that primarily contributed to model prediction accuracy in women. In stark contrast to this finding, ‘classic’ personality, mental health, and neurocognitive factors that have often been linked to chronic cannabis use and dependence in previous studies were primarily driving effects in men. In particular, the ‘male-dominated’ factors included the personality trait openness, a history of conduct disorder, externalizing symptoms, and working memory performance. For brain factors, there were both ‘female-dominated’ factors, such as a smaller right superior temporal area (which was not among the highest ranked overall factors), and ‘male-dominated’ factors, such as low hippocampal volume, higher postcentral thickness and lower global efficiency of the precentral gyrus in the somatosensory-motor system. A smaller right superior temporal regions, the ‘female-dominated’ brain factor, has been previously observed in adolescent cannabis users^94^, and is assumed to underlie social perception^95^, consistent with the greater importance of environmental factors such as social support in women. Reduced hippocampal volume, a ‘male-dominated’ brain factor, is probably the most commonly reported brain structural abnormality in CUD^96^, and may be linked to ‘male-dominated’ impaired working memory performance^97^. Increased postcentral cortical thickness, another ‘male-dominated’ brain factor, has been shown to correlate with earlier age of onset of cannabis use in young adults^98^, and may be a marker of altered somatosensory processing as a consequence of cannabis use^99^. Finally, abnormalities in precentral gyrus function, the third ‘male-dominated’ brain factor that we identified, has been previously observed in young adults with cannabis use^98,100^, and are assumed to play a role in response inhibition of motor impulses (e.g. lack of self-regulation as evidenced by increased externalizing symptoms in men)^98^. Taken together, these results suggest that environmental factors (educational attainment, instrumental support) and their associated brain correlates play a larger role in women, and the ‘classic’ individual factors that have been most often linked to cannabis addiction and their associated brain correlates, contribute more strongly to CUD in men.

A limitation of the current study is the relatively small number of women included in some of the models. However, the current results provide compelling initial evidence for sex/gender differences in the multifactorial factors underlying CUD in adults, which had not been previously investigated using a multi-domain approach. Strikingly, these results closely mirror previous findings from a machine learning analysis that investigated predictors of onset of cannabis use in adolescence. Spechler and colleagues (2019) found that individual level factors such as sensation/novelty seeking were predictive of cannabis use onset in boys, whereas factors that are more closely linked to the environment, such as verbal IQ, sexual relationships and parent personality, were predictive in girls^10^. These findings also fit with our recent review and empirical data demonstrating a much greater importance of social support as a protective factor preventing the escalation of alcohol use in adolescence and maintenance of alcohol misuse in adulthood particularly in girls and women, as compared to boys and men^101^. We are only aware of one previous study on sex/gender differences in CUD in adults^102^. This study specifically investigated sex/gender differences in the role of social support and found a stronger protective relationship of social support in women as compared to men^102^. Additionally, our results extend previous findings on cannabis use in adolescence that suggest a stronger influence of environmental factors in girls as compared to boys^103–106^. A twin study found that the overall contribution of environmental factors for predicting cannabis use levels, as compared to individual predictive factors, was larger in adolescent girls versus boys^103^. Similarly, a longitudinal study described that environmental influences such as attending public (versus private) schools, academic performance, living in a single-parent family, spending time in bars/discos and drug use among friends had a stronger influence on cannabis use levels in adolescent girls as compared to boys^104^. The same study found that individual factors such as prior history of smoking/alcohol consumption and antisocial behavior were stronger predictors in adolescent boys^104^. Furthermore, one study demonstrated that a protective family environment had a stronger influence on cannabis use onset in adolescent girls as compared to boys^105^, and that higher life satisfaction was a stronger protective factor against frequent cannabis use among adolescent girls than boys^106^. Overall, the resemblance of the general pattern of a stronger influence of environmental versus individual factors in girls and women is striking and warrants further investigation.

## Conclusion

Our data-driven investigation of the factors linked to CUD in young adults in the United States revealed a small number of environmental, personality, mental health, neurocognitive and brain factors that were consistently linked to high cannabis use levels and dependence. The importance of these factors in classifying high use levels and dependence varied by sex/gender. Environmental factors contributed more strongly to CUD in women, whereas individual factors, such as personality, mental health and neurocognitive factors, had a larger importance in men. The current findings therefore warrant further investigations into sex/gender differences in young adults with CUD, and suggest the importance of understanding how these differences may inform the development of sex/gender-specific treatment approaches for addiction medicine.

## Supplementary Information

Supplementary Information.

## Data Availability

All data used in the present study are available for download from the Human Connectome Project (www.humanconnectome.org). Users must agree to data use terms for the HCP before being allowed access to the data and ConnectomeDB, details are provided at https://www.humanconnectome.org/study/hcp-young-adult/data-use-terms. The HCP has implemented a two-tiered plan for data sharing, with different provisions for handling Open Access data and Restricted data (e.g., data related to substance use). See https://www.humanconnectome.org/study/hcp-young-adult/document/restricted-data-usage for more details. Users must also consult with their local IRB or Ethics Committee (EC) before utilizing the HCP data to ensure that IRB or EC approval is not needed before beginning research with the HCP data. If needed, and upon request, the HCP will provide a certificate to users confirming acceptance of the HCP Open and Restricted Access Data Use Terms. See https://www.humanconnectome.org/study/hcp-young-adult/data-use-terms.

## Code availability

The code for preprocessing the data and all computational models are available on https://github.com/explainable-cannabis/explainable-cannabis-paper.
