# Supplementary material for: Explainable machine learning analysis reveals sex and gender differences in the phenotypic and neurobiological markers of Cannabis Use Disorder
Source: Sci Rep. 2022 Sep 17;12:15624. doi: 10.1038/s41598-022-19804-2 (PMC9482622; doi:10.1038/s41598-022-19804-2)
Supplement: Supplementary file 1 — Supplementary Information. [file 41598_2022_19804_MOESM1_ESM.pdf]

SUPPLEMENT for:

Explainable Machine Learning Analysis Reveals Sex and Gender Differences in the Phenotypic and Neurobiological Markers of Cannabis Use Disorder

Authors: Gregory R. Niklason[1], Eric Rawls[1], Sisi Ma[2], Erich Kummerfeld[2], Andrea M. Maxwell[3], Leyla R. Brucar[1], Gunner Drossel[4], and Anna Zilverstand[1,5*]

Affiliations: [1]Department of Psychiatry and Behavioral Sciences, [2]Institute for Health Informatics, [3]Medical Scientist Training Program, [4]Graduate Program in Neuroscience, [5]Medical Discovery Team on Addiction; all at University of Minnesota, Minneapolis, MN, USA.

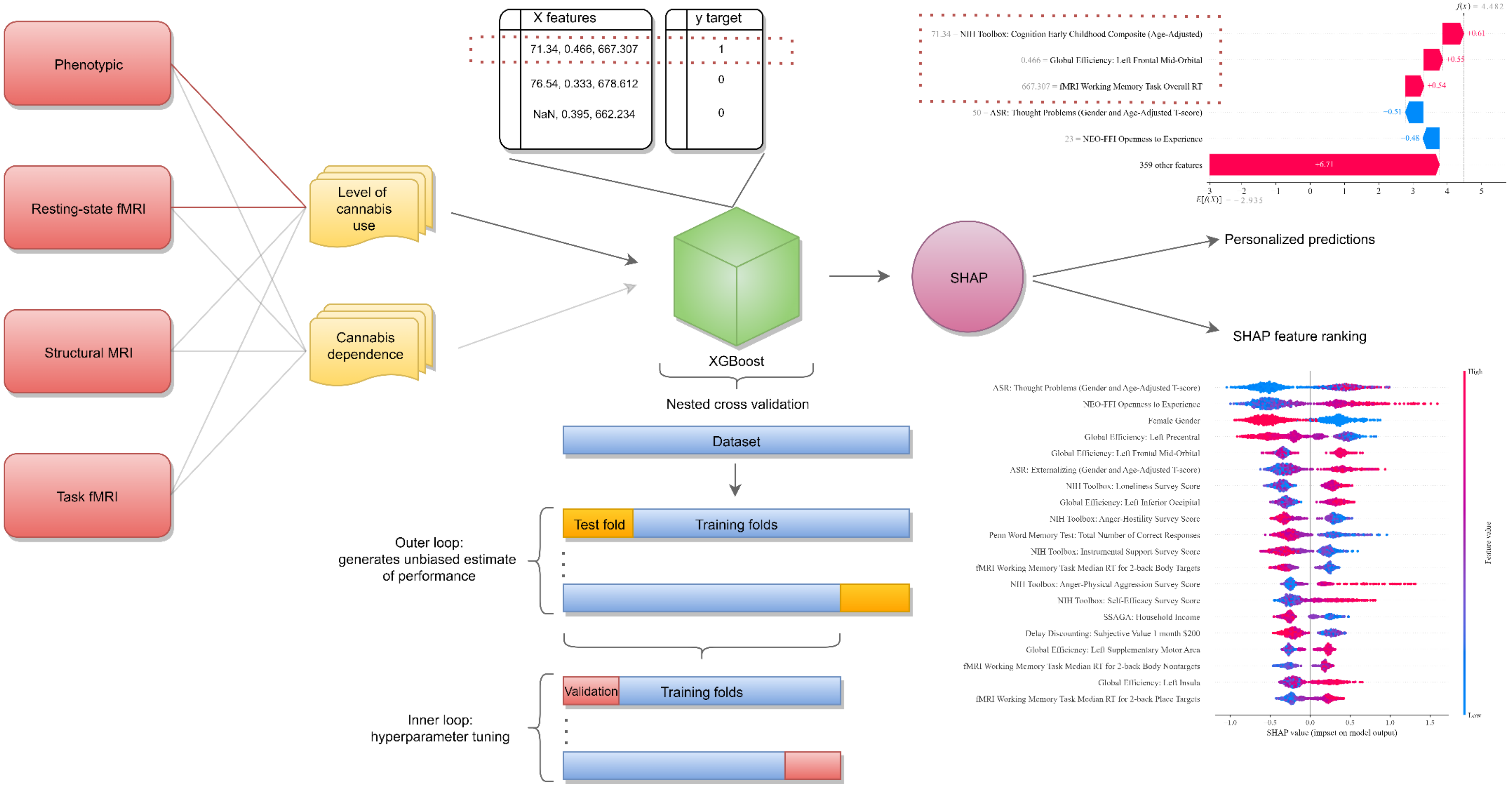

Supplementary Figure 1: Data Analysis Flow. All available phenotypic and neuroimaging data was included. To compare classification accuracy across data modalities, we built one model per modality and outcome: 1) cannabis use levels and 2) cannabis dependence. We used XGBoost, a tree based ensemble machine learning algorithm and nested cross-validation to tune hyperparameters (inner loop) and evaluate classification performance (outer loop), generating an unbiased estimate of model performance. A novel ranking tool, SHapley's Additive exPlanations (SHAP), was employed to compute the unique and additive importance of each model feature (factor), generating personalized predictions on an individual level and computing average SHAP feature value rankings on a group level.

Supplementary Table 1. Complete list of included phenotypic variables.

	HCP Database Variable Name	Description
0	MMSE_Score	Mini Mental Status Exam Total Score
1	PSQI_Score	PSQI: Sleep Total Score
2	PSQI_Comp1	PSQI: Sleep Component 1 Score
3	PSQI_Comp2	PSQI: Sleep Component 2 Score
4	PSQI_Comp3	PSQI: Sleep Component 3 Score
5	PSQI_Comp4	PSQI: Sleep Component 4 Score
6	PSQI_Comp5	PSQI: Sleep Component 5 Score
7	PSQI_Comp6	PSQI: Sleep Component 6 Score
8	PSQI_Comp7	PSQI: Sleep Component 7 Score
9	PSQI_Min2Asleep	PSQI: Minutes to fall asleep (past month)
10	PSQI_AmtSleep	PSQI: Hours of sleep per night (past month)
11	PSQI_Latency30Min	PSQI: Sleep Trouble - Can't get to sleep within 30 minutes
12	PSQI_WakeUp	PSQI: Sleep Trouble - Wake up in middle of night or early morning
13	PSQI_Bathroom	PSQI: Sleep Trouble - Get up to use bathroom
14	PSQI_Breathe	PSQI: Sleep Trouble - Can't breathe comfortably
15	PSQI_Snore	PSQI: Sleep Trouble - Cough or snore loudly
16	PSQI_TooCold	PSQI: Sleep Trouble - Feel too cold
17	PSQI_TooHot	PSQI: Sleep Trouble - Feel too hot
18	PSQI_BadDream	PSQI: Sleep Trouble - Had bad dreams
19	PSQI_Pain	PSQI: Sleep Trouble - Have pain
20	PSQI_Other	PSQI: Sleep Trouble - Other
21	PSQI_Quality	PSQI: Describe overall sleep quality
22	PSQI_SleepMeds	PSQI: How often taken sleep medicine
23	PSQI_DayStayAwake	PSQI: How often trouble staying awake
24	PSQI_DayEnthusiasm	PSQI: How often trouble keeping up enthusiasm
25	PSQI_BedPtnrRmate	PSQI: Have bed partner or roommate
26	PSQI_GetUpTime	PSQI: Time get up in morning (past month)
27	PSQI_BedTime	PSQI: Usual bed time (past month)
28	PicSeq_AgeAdj	NIH Toolbox: Picture Sequence Memory Test (Age-Adjusted)
29	CardSort_AgeAdj	NIH Toolbox: Dimensional Change Card Sort (Age-Adjusted)
30	Flanker_AgeAdj	NIH Toolbox: Flanker Inhibitory Control and Attention Test (Age-Adjusted)
31	PMAT24_A_CR	Penn Progressive Matrices: Number of Correct Responses
32	PMAT24_A_SI	Penn Progressive Matrices: Total Skipped Items
33	PMAT24_A_RTCR	Penn Progressive Matrices: Median RT Correct Responses
34	ReadEng_AgeAdj	NIH Toolbox: Oral Reading Recognition Test (Age-Adjusted)
35	PicVocab_AgeAdj	NIH Toolbox: Picture Vocabulary Test (Age-Adjusted)
36	ProcSpeed_AgeAdj	NIH Toolbox: Processing Speed (Age-Adjusted)
37	DDisc_SV_1mo_200	Delay Discounting: Subjective Value 1 month $200
38	DDisc_SV_6mo_200	Delay Discounting: Subjective Value 6 months $200
39	DDisc_SV_1yr_200	Delay Discounting: Subjective Value 1 year $200
40	DDisc_SV_3yr_200	Delay Discounting: Subjective Value 3 years $200
41	DDisc_SV_5yr_200	Delay Discounting: Subjective Value 5 years $200
42	DDisc_SV_10yr_200	Delay Discounting: Subjective Value 10 years $200
43	DDisc_SV_1mo_40K	Delay Discounting: Subjective Value 1 month $40K

44	DDisc_SV_6mo_40K	Delay Discounting: Subjective Value 6 months $40K
45	DDisc_SV_1yr_40K	Delay Discounting: Subjective Value 1 year $40K
46	DDisc_SV_3yr_40K	Delay Discounting: Subjective Value 3 years $40K
47	DDisc_SV_5yr_40K	Delay Discounting: Subjective Value 5 years $40K
48	DDisc_SV_10yr_40K	Delay Discounting: Subjective Value 10 years $40K
49	DDisc_AUC_200	Delay Discounting: Area Under the Curve $200
50	DDisc_AUC_40K	Delay Discounting: Area Under the Curve $40K
51	VSPLOT_TC	Variable Short Penn Line Orientation: Total Number Correct
52	VSPLOT_CRTE	Variable Short Penn Line Orientation: Median RT/Expected Number of Clicks Correct
53	VSPLOT_OFF	Variable Short Penn Line Orientation: Total Positions Off for All Trials
54	SCPT_TP	Short Penn Continuous Performance Test: True Positives
55	SCPT_TN	Short Penn Continuous Performance Test: True Negatives
56	SCPT_FP	Short Penn Continuous Performance Test: False Positives
57	SCPT_FN	Short Penn Continuous Performance Test: False Negatives
58	SCPT_TPRT	Short Penn Continuous Performance Test: Median RT True Positives
59	SCPT_SEN	Short Penn Continuous Performance Test: Sensitivity
60	SCPT_SPEC	Short Penn Continuous Performance Test: Specificity
61	SCPT_LRNR	Short Penn Continuous Performance Test: Longest Run of Non-Responses
62	IWRD_TOT	Penn Word Memory Test: Total Number of Correct Responses
63	IWRD_RTC	Penn Word Memory Test: Median RT Correct Responses
64	ListSort_AgeAdj	NIH Toolbox: List Sorting Working Memory Test (Age-Adjusted)
65	CogFluidComp_AgeAdj	NIH Toolbox: Cognition Fluid Composite (Age-Adjusted)
66	CogEarlyComp_AgeAdj	NIH Toolbox: Cognition Early Childhood Composite (Age-Adjusted)
67	CogTotalComp_AgeAdj	NIH Toolbox: Cognition Total Composite Score (Age-Adjusted)
68	CogCrystalComp_AgeAdj	NIH Toolbox: Cognition Crystallized Composite (Age-Adjusted)
69	ER40_CR	Penn Emotion Recognition Test: Number of Correct Responses
70	ER40_CRT	Penn Emotion Recognition Test: Correct Responses Median RT (ms)
71	ER40ANG	Penn Emotion Recognition Test: Number of Correct Anger
72	ER40FEAR	Penn Emotion Recognition Test: Number of Correct Fear
73	ER40HAP	Penn Emotion Recognition Test: Number of Correct Happy
74	ER40NOE	Penn Emotion Recognition Test: Number of Correct Neutral
75	ER40SAD	Penn Emotion Recognition Test: Number of Correct Sad
76	AngAffect_Unadj	NIH Toolbox: Anger-Affect Survey Score
77	AngHostil_Unadj	NIH Toolbox: Anger-Hostility Survey Score
78	AngAggr_Unadj	NIH Toolbox: Anger-Physical Aggression Survey Score
79	FearAffect_Unadj	NIH Toolbox: Fear-Affect Arousal Survey Score
80	FearSomat_Unadj	NIH Toolbox: Fear-Somatic Arousal Survey Score
81	Sadness_Unadj	NIH Toolbox: Sadness Survey Score
82	LifeSatisf_Unadj	NIH Toolbox: General Life Satisfaction Survey Score
83	MeanPurp_Unadj	NIH Toolbox: Meaning and Purpose Survey Score
84	PosAffect_Unadj	NIH Toolbox: Positive Affect Survey Score
85	Friendship_Unadj	NIH Toolbox: Friendship Survey Score
86	Loneliness_Unadj	NIH Toolbox: Loneliness Survey Score
87	PercHostil_Unadj	NIH Toolbox: Perceived Hostility Survey Score
88	PercReject_Unadj	NIH Toolbox: Perceived Rejection Survey Score
89	EmotSupp_Unadj	NIH Toolbox: Emotional Support Survey Score

90	InstruSupp_Unadj	NIH Toolbox: Instrumental Support Survey Score
91	PercStress_Unadj	NIH Toolbox: Perceived Stress Survey Score
92	SelfEff_Unadj	NIH Toolbox: Self-Efficacy Survey Score
93	Emotion_Task_Acc	fMRI OVERALL Emotion Task Accuracy
94	Emotion_Task_Median_RT	fMRI OVERALL Emotion Task RT
95	Emotion_Task_Face_Acc	fMRI Emotion Task FACE Accuracy
96	Emotion_Task_Face_Median_RT	fMRI Emotion Task FACE Median RT
97	Emotion_Task_Shape_Acc	fMRI Emotion Task SHAPE Accuracy
98	Emotion_Task_Shape_Median_RT	fMRI Emotion Task SHAPE Median RT
99	Gambling_Task_Perc_Larger	fMRI Gambling Task Overall Percentage 'Larger'
100	Gambling_Task_Perc_Smaller	fMRI Gambling Task Overall Percentage 'Smaller'
101	Gambling_Task_Perc_NLR	fMRI Gambling Task Overall Percentage No Logged Response
102	Gambling_Task_Median_RT_Larger	fMRI Gambling Task Overall RT 'Larger'
103	Gambling_Task_Median_RT_Smaller	fMRI Gambling Task Overall RT 'Smaller'
104	Gambling_Task_Reward_Perc_Larger	fMRI Gambling Task Percentage 'Larger' in Reward
105	Gambling_Task_Reward_Median_RT_Larger	fMRI Gambling Task Median RT 'Larger' in Reward
106	Gambling_Task_Reward_Perc_Smaller	fMRI Gambling Task Percentage 'Smaller' in Reward
107	Gambling_Task_Reward_Median_RT_Smaller	fMRI Gambling Task Median RT 'Smaller' in Reward
108	Gambling_Task_Reward_Perc_NLR	fMRI Gambling Task Percentage No Logged Response in Reward
109	Gambling_Task_Punish_Perc_Larger	fMRI Gambling Task Percentage 'Larger' in Punish
110	Gambling_Task_Punish_Median_RT_Larger	fMRI Gambling Task Median RT 'Larger' in Punish
111	Gambling_Task_Punish_Perc_Smaller	fMRI Gambling Task Percentage 'Smaller' in Punish
112	Gambling_Task_Punish_Median_RT_Smaller	fMRI Gambling Task Median RT 'Smaller' in Punish
113	Gambling_Task_Punish_Perc_NLR	fMRI Gambling Task Percentage No Logged Response in Punish
114	Language_Task_Acc	fMRI Language Task OVERALL Accuracy
115	Language_Task_Median_RT	fMRI Language Task OVERALL Median RT
116	Language_Task_Story_Acc	fMRI Language Task STORY Accuracy
117	Language_Task_Story_Median_RT	fMRI Language Task STORY Median RT
118	Language_Task_Story_Avg_Difficulty_Level	fMRI Language Task STORY Difficulty Level
119	Language_Task_Math_Acc	fMRI Language Task MATH Accuracy
120	Language_Task_Math_Median_RT	fMRI Language Task MATH Median RT
121	Language_Task_Math_Avg_Difficulty_Level	fMRI Language Task MATH Difficulty Level
122	Relational_Task_Acc	fMRI Relational Task OVERALL Accuracy
123	Relational_Task_Median_RT	fMRI Relational Task OVERALL RT
124	Relational_Task_Match_Acc	fMRI Relational Task MATCH Accuracy
125	Relational_Task_Match_Median_RT	fMRI Relational Task MATCH Median RT
126	Relational_Task_Rel_Acc	fMRI Relational Task RELATIONAL block (REL) Accuracy
127	Relational_Task_Rel_Median_RT	fMRI Relational Task RELATIONAL block (REL) Median RT
128	Social_Task_Perc_Random	fMRI Social Task Overall Percentage 'Random'
129	Social_Task_Perc_TOM	fMRI Social Task Overall Percentage 'TOM'
130	Social_Task_Perc_Unsure	fMRI Social Task Overall Percentage 'Unsure'
131	Social_Task_Perc_NLR	fMRI Social Task Overall Percentage No Logged Response
132	Social_Task_Median_RT_Random	fMRI Social Task Overall RT 'Random'
133	Social_Task_Median_RT_TOM	fMRI Social Task Overall RT 'TOM'
134	Social_Task_Median_RT_Unsure	fMRI Social Task Overall RT 'Unsure'

135	Social_Task_Random_Perc_Random	fMRI Social Task Percentage 'Random' in Random condition
136	Social_Task_Random_Median_RT_Random	fMRI Social Task Median RT 'Random' in Random condition
137	Social_Task_Random_Perc_TOM	fMRI Social Task Percentage 'TOM' in Random condition
138	Social_Task_Random_Median_RT_TOM	fMRI Social Task Median RT 'TOM' in Random condition
139	Social_Task_Random_Perc_Unsure	fMRI Social Task Percentage 'Unsure' in Random Condition
140	Social_Task_Random_Median_RT_Unsure	fMRI Social Task Median RT 'Unsure' in Random condition
141	Social_Task_Random_Perc_NLR	fMRI Social Task Percentage No Logged Response in Random condition
142	Social_Task_TOM_Perc_Random	fMRI Social Task Percentage 'Random' in Social (TOM) condition
143	Social_Task_TOM_Median_RT_Random	fMRI Social Task Median RT 'Random' in Social (TOM) condition
144	Social_Task_TOM_Perc_TOM	fMRI Social Task Percentage 'TOM' in Social (TOM) condition
145	Social_Task_TOM_Median_RT_TOM	fMRI Social Task Median RT 'TOM' in Social (TOM) condition
146	Social_Task_TOM_Perc_Unsure	fMRI Social Task Percentage 'Unsure' in Social (TOM) condition
147	Social_Task_TOM_Median_RT_Unsure	fMRI Social Task Median RT 'Unsure' in Social (TOM) condition
148	Social_Task_TOM_Perc_NLR	fMRI Social Task Percentage No Logged Response in Social (TOM) condition
149	WM_Task_Acc	fMRI Working Memory Task Overall Accuracy
150	WM_Task_Median_RT	fMRI Working Memory Task Overall RT
151	WM_Task_2bk_Acc	fMRI Working Memory Task Accuracy for 2-back
152	WM_Task_2bk_Median_RT	fMRI Working Memory Task Median RT for 2-back
153	WM_Task_0bk_Acc	fMRI Working Memory Task Accuracy for 0-back
154	WM_Task_0bk_Median_RT	fMRI Working Memory Task Median RT for 0-back
155	WM_Task_0bk_Body_Acc	fMRI Working Memory Task Accuracy for 0-back Body
156	WM_Task_0bk_Body_Acc_Target	fMRI Working Memory Task Accuracy for 0-back Body Targets
157	WM_Task_0bk_Body_Acc_Nontarget	fMRI Working Memory Task Accuracy for 0-back Body Nontargets
158	WM_Task_0bk_Face_Acc	fMRI Working Memory Task Accuracy for 0-back Face
159	WM_Task_0bk_Face_Acc_Target	fMRI Working Memory Task Accuracy for 0-back Face Targets
160	WM_Task_0bk_Face_ACC_Nontarget	fMRI Working Memory Task Accuracy for 0-back Face Nontargets
161	WM_Task_0bk_Place_Acc	fMRI Working Memory Task Accuracy for 0-back Place
162	WM_Task_0bk_Place_Acc_Target	fMRI Working Memory Task Accuracy for 0-back Place Targets
163	WM_Task_0bk_Place_Acc_Nontarget	fMRI Working Memory Task Accuracy for 0-back Place Nontargets
164	WM_Task_0bk_Tool_Acc	fMRI Working Memory Task Accuracy for 0-back Tool
165	WM_Task_0bk_Tool_Acc_Target	fMRI Working Memory Task Accuracy for 0-back Tool Targets
166	WM_Task_0bk_Tool_Acc_Nontarget	fMRI Working Memory Task Accuracy for 0-back Tool Nontargets
167	WM_Task_2bk_Body_Acc	fMRI Working Memory Task Accuracy for 2-back Body
168	WM_Task_2bk_Body_Acc_Target	fMRI Working Memory Task Accuracy for 2-back Body Targets
169	WM_Task_2bk_Body_Acc_Nontarget	fMRI Working Memory Task Accuracy for 2-back Body Nontargets
170	WM_Task_2bk_Face_Acc	fMRI Working Memory Task Accuracy for 2-back Face
171	WM_Task_2bk_Face_Acc_Target	fMRI Working Memory Task Accuracy for 2-back Face Targets
172	WM_Task_2bk_Face_Acc_Nontarget	fMRI Working Memory Task Accuracy for 2-back Face Nontargets
173	WM_Task_2bk_Place_Acc	fMRI Working Memory Task Accuracy for 2-back Place
174	WM_Task_2bk_Place_Acc_Target	fMRI Working Memory Task Accuracy for 2-back Place Targets
175	WM_Task_2bk_Place_Acc_Nontarget	fMRI Working Memory Task Accuracy for 2-back Place Nontargets
176	WM_Task_2bk_Tool_Acc	fMRI Working Memory Task Accuracy for 2-back Tool
177	WM_Task_2bk_Tool_Acc_Target	fMRI Working Memory Task Accuracy for 2-back Tool Targets
178	WM_Task_2bk_Tool_Acc_Nontarget	fMRI Working Memory Task Accuracy for 2-back Tool Nontargets
179	WM_Task_0bk_Body_Median_RT	fMRI Working Memory Task Median RT for 0-back Body
180	WM_Task_0bk_Body_Median_RT_Target	fMRI Working Memory Task Median RT for 0-back Body Targets

181	WM_Task_0bk_Body_Median_RT_Nontarget	fMRI Working Memory Task Median RT for 0-back Body Nontargets
182	WM_Task_0bk_Face_Median_RT	fMRI Working Memory Task Median RT for 0-back Face
183	WM_Task_0bk_Face_Median_RT_Target	fMRI Working Memory Task Median RT for 0-back Face Targets
184	WM_Task_0bk_Face_Median_RT_Nontarget	fMRI Working Memory Task Median RT for 0-back Face Nontargets
185	WM_Task_0bk_Place_Median_RT	fMRI Working Memory Task Median RT for 0-back Place
186	WM_Task_0bk_Place_Median_RT_Target	fMRI Working Memory Task Median RT for 0-back Place Targets
187	WM_Task_0bk_Place_Median_RT_Nontarget	fMRI Working Memory Task Median RT for 0-back Place Nontargets
188	WM_Task_0bk_Tool_Median_RT	fMRI Working Memory Task Median RT for 0-back Tool
189	WM_Task_0bk_Tool_Median_RT_Target	fMRI Working Memory Task Median RT for 0-back Tool Targets
190	WM_Task_0bk_Tool_Median_RT_Nontarget	fMRI Working Memory Task Median RT for 0-back Tool Nontargets
191	WM_Task_2bk_Body_Median_RT	fMRI Working Memory Task Median RT for 2-back Body
192	WM_Task_2bk_Body_Median_RT_Target	fMRI Working Memory Task Median RT for 2-back Body Targets
193	WM_Task_2bk_Body_Median_RT_Nontarget	fMRI Working Memory Task Median RT for 2-back Body Nontargets
194	WM_Task_2bk_Face_Median_RT	fMRI Working Memory Task Median RT for 2-back Face
195	WM_Task_2bk_Face_Median_RT_Target	fMRI Working Memory Task Medain RT for 2-back Face Targets
196	WM_Task_2bk_Face_Median_RT_Nontarget	fMRI Working Memory Task Median RT for 2-back Face NonTargets
197	WM_Task_2bk_Place_Median_RT	fMRI Working Memory Task Median RT for 2-back Place
198	WM_Task_2bk_Place_Median_RT_Target	fMRI Working Memory Task Median RT for 2-back Place Targets
199	WM_Task_2bk_Place_Median_RT_Nontarget	fMRI Working Memory Task Median RT for 2-back Place Nontargets
200	WM_Task_2bk_Tool_Median_RT	fMRI Working Memory Task Median RT for 2-back Tool
201	WM_Task_2bk_Tool_Median_RT_Target	fMRI Working Memory Task Median RT for 2-back Tool Targets
202	WM_Task_2bk_Tool_Median_RT_Nontarget	fMRI Working Memory Task Median RT for 2-back Tool Nontargets
203	NEOFAC_A	NEO-FFI Agreeableness
204	NEOFAC_O	NEO-FFI Openness to Experience
205	NEOFAC_C	NEO-FFI Conscientiousness
206	NEOFAC_N	NEO-FFI Neuroticism
207	NEOFAC_E	NEO-FFI Extraversion
208	Age_in_Yrs	Age in Years
209	Gender_F	Female Gender
210	Race_Am. Indian/Alaskan Nat.	Race: Am. Indian/Alaskan Nat.
211	Race_Asian/Nat. Hawaiian/Othr Pacific Is.	Race: Asian/Nat. Hawaiian/Other Pacific Is.
212	Race_Black or African Am.	Race: Black/African American
213	Race_More than one	Race: More than one
214	Race_Unknown or Not Reported	Race: Unknown/Not Reported
215	Race_White	Race: White
216	Ethnicity_Hispanic/Latino	Ethnicity: Hispanic/Latino
217	Ethnicity_Not Hispanic/Latino	Ethnicity: Not Hispanic/Latino
218	Ethnicity_Unknown or Not Reported	Ethnicity: Unknown/Not Reported
219	ZygositySR_	ZygositySR: Missing
220	ZygositySR_MZ	ZygositySR: MZ
221	ZygositySR_NotMZ	ZygositySR: Not MZ
222	ZygositySR_NotTwin	ZygositySR: Not Twin
223	ZygosityGT_	ZygosityGT: Missing
224	ZygosityGT_DZ	ZygosityGT: DZ

225	ZygosityGT_MZ	ZygosityGT: MZ
226	TestRetestInterval	Test Retest Interval
227	Handedness	Handedness
228	SSAGA_Employ	SSAGA: Employment Status
229	SSAGA_Income	SSAGA: Household Income
230	SSAGA_Educ	SSAGA: Education
231	SSAGA_InSchool	SSAGA: Still in School
232	SSAGA_Rlshp	SSAGA: Relationship Status
233	SSAGA_MOBorn	SSAGA: Missouri Born
234	FamHist_Moth_Scz	Mother Schizophrenia or Psychosis
235	FamHist_Fath_Scz	Father Schizophrenia or Psychosis
236	FamHist_Moth_Dep	Mother Depression
237	FamHist_Fath_Dep	Father Depression
238	FamHist_Moth_BP	Mother Bipolar Disorder
239	FamHist_Fath_BP	Father Bipolar Disorder
240	FamHist_Moth_Anx	Mother Anxiety Needing Treatment
241	FamHist_Fath_Anx	Father Anxiety Needing Treatment
242	FamHist_Moth_DrgAlc	Mother Drug or Alcohol Problems
243	FamHist_Fath_DrgAlc	Father Drug or Alcohol Problems
244	FamHist_Moth_Alz	Mother Alzheimer's or Dementia
245	FamHist_Fath_Alz	Father Alzheimer's or Dementia
246	FamHist_Moth_PD	Mother Parkinson's Disease
247	FamHist_Fath_PD	Father Parkinson's Disease
248	FamHist_Moth_TS	Mother Tourette's Syndrome
249	FamHist_Fath_TS	Father Tourette's Syndrome
250	FamHist_Moth_None	Mother None of the Above
251	FamHist_Fath_None	Father None of the Above
252	ASR_Anxd_Pct	ASR: Anxious/Depressed (Gender and Age-Adjusted Percentile)
253	ASR_Witd_T	ASR: Withdrawn (Gender and Age-Adjusted T-score)
254	ASR_Soma_T	ASR: Somatic Complaints (Gender and Age-Adjusted T-score)
255	ASR_Thot_T	ASR: Thought Problems (Gender and Age-Adjusted T-score)
256	ASR_Attn_T	ASR: Attention Problems (Gender and Age-Adjusted T-score)
257	ASR_Aggr_T	ASR: Aggressive Behavior (Gender and Age-Adjusted T-score)
258	ASR_Intr_T	ASR: Intrusive (Gender and Age-Adjusted T-score)
259	ASR_Intn_T	ASR: Internalizing (Gender and Age-Adjusted T-score)
260	ASR_Extn_T	ASR: Externalizing (Gender and Age-Adjusted T-score)
261	ASR_TAO_Sum	ASR: Sum of Thought, Attention, and Other Problems Raw Score
262	ASR_Totp_T	ASR: Total Problems (Gender and Age-Adjusted T-score)
263	DSM_Depr_T	ASR DSM: Depressive Problems (Gender and Age-Adjusted T-score)
264	DSM_Anxi_T	ASR DSM: Anxiety Problems (Gender and Age-Adjusted T-score)
265	DSM_Somp_T	ASR DSM: Somatic Problems (Gender and Age-Adjusted T-score)
266	DSM_Avoid_T	ASR DSM: Avoidant Personality Problems (Gender and Age-Adjusted T-score)
267	DSM_Adh_T	ASR DSM: AD/H Problems (Gender and Age-Adjusted T-score)
268	DSM_Antis_T	ASR DSM: Antisocial Personality Problems (Gender and Age-Adjusted T-score)
269	SSAGA_ChildhoodConduct	SSAGA: Childhood Conduct Problems
270	SSAGA_PanicDisorder	SSAGA: Panic Disorder

271	SSAGA_Agoraphobia	SSAGA: Agoraphobia
272	SSAGA_Depressive_Ep	SSAGA: Major Depressive Episode
273	SSAGA_Depressive_Sx	SSAGA: Number of Depressive Symptoms

Supplementary Table 2. Task fMRI COPE (Coefficient of Parameter Estimates) Contrasts Included.

Task Name	Task Number	Contrast Number	Contrast Name
Working Memory (N-back)	1	11	2BK-0BK
Gambling	2	1	PUNISH
Gambling	2	2	REWARD
Motor	3	2	LF
Motor	3	3	LH
Motor	3	4	RF
Motor	3	5	RH
Motor	3	6	T
Language	4	4	STORY-MATH
Social Cognition	5	6	TOM-RANDOM
Relational	6	4	REL-MATCH
Emotion	7	3	FACES-SHAPES

Supplementary Table 3. Best performing hyperparameters for each model.

	Learning rate	Max depth	Subsample
Phenotypic+FreeSurfer 1+	0.01	10	0.6
Phenotypic+FreeSurfer 10+	0.20	8	0.8
Phenotypic+FreeSurfer 100+	0.01	12	0.6
Phenotypic+FreeSurfer 1000+	0.02	6	0.6
Phenotypic+FreeSurfer Dependence	0.05	8	1.0
Phenotypic+Global Efficiency 1+	0.01	12	0.6
Phenotypic+Global Efficiency 10+	0.01	8	1.0
Phenotypic+Global Efficiency 100+	0.20	4	0.6
Phenotypic+Global Efficiency 1000+	0.10	6	1.0
Phenotypic+Global Efficiency Dependence	0.10	8	0.8

Supplementary Table 4. SHAP factor ranking for the two bimodal classification models.

Factor	Rank 1+	Rank 10+	Rank 100+	Rank 1000+	Rank CUD	Median Rank
Phenotypic + Freesurfer Model						
NEO-FFI Openness	1	1	1	1	1	1
ASR Externalizing	2	6	2	2	70	2
Freesurfer: Left Hippocampus Volume	4	3	8	52	4	4
Female Gender	89	2	3	4	7	4
NIH Toolbox Anger Aggression	7	4	6	8	75	7
Freesurfer: Brainstem Volume	6	5	110	48	8	8
Freesurfer: CSF Volume	8	11	10	10	110	10
Freesurfer: Left Frontal Pole Thickness	44	9	14	6	87	14
fMRI N-Back Task RT: 2-back place target	12	119	15	14	53	15
fMRI N-Back Task RT: 2-back body target	10	16	20	16	22	16
SSAGA Income	270	12	5	19	17	17
SSAGA Childhood Conduct	18	21	17	12	2	17
NIH Toolbox Picture Vocabulary (age-adjusted)	13	32	19	13	41	19
Phenotypic + Global Efficiency Model						
NEO-FFI Openness	1	1	1	2	1	1
ASR Externalizing	2	3	4	6	35	4
Female Gender	121	4	3	3	5	4
fMRI N-Back Task RT: 2-back body target	5	8	6	12	18	8
NIH Toolbox Picture Vocabulary (age-adjusted)	8	6	7	42	20	8
NIH Toolbox Anger Aggression	6	2	13	13	27	13
SSAGA Education	246	14	2	33	2	14
SSAGA Income	165	11	8	15	52	15
Global Efficiency: Left Rolandic Operculum	10	13	15	32	90	15
fMRI N-Back Task RT: 2-back body nontarget	102	16	10	18	85	18
Global Efficiency: Left Insula	9	18	105	19	151	19
Global Efficiency: Left Superior Occipital	19	12	32	25	11	19
fMRI N-Back Task RT: 2-back place target	11	75	12	20	40	20

Only factors with a median rank of 20 or less (i.e., the highest ranked among the total included >1000 features) are presented.

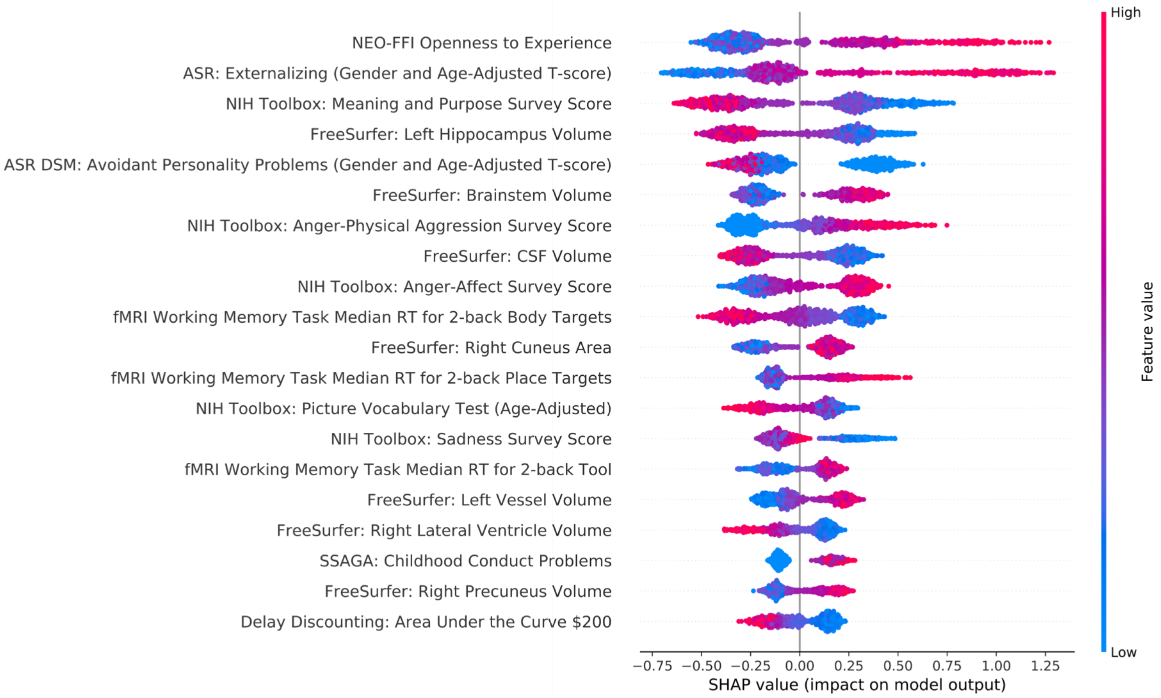

Supplementary Figure 2. *SHAP factor ranking for the phenotypic+Freesurfer model classifying 1+ lifetime cannabis use. More positive SHAP values on the x-axis indicate that the observed factor pushed the classification closer towards cannabis use, whereas more negative SHAP values indicate that the factor pushed the classification away from cannabis use. Factors are ranked in order of greatest average SHAP value, which indicates the importance of that factor. Individual points represent the model output for each individual in the sample. The position of a dot on the x-axis represents the impact of the observed factor on the model output for the individual. The color of the individual dots represent the value of the observed measurements, with blue indicating lower and red higher values.*

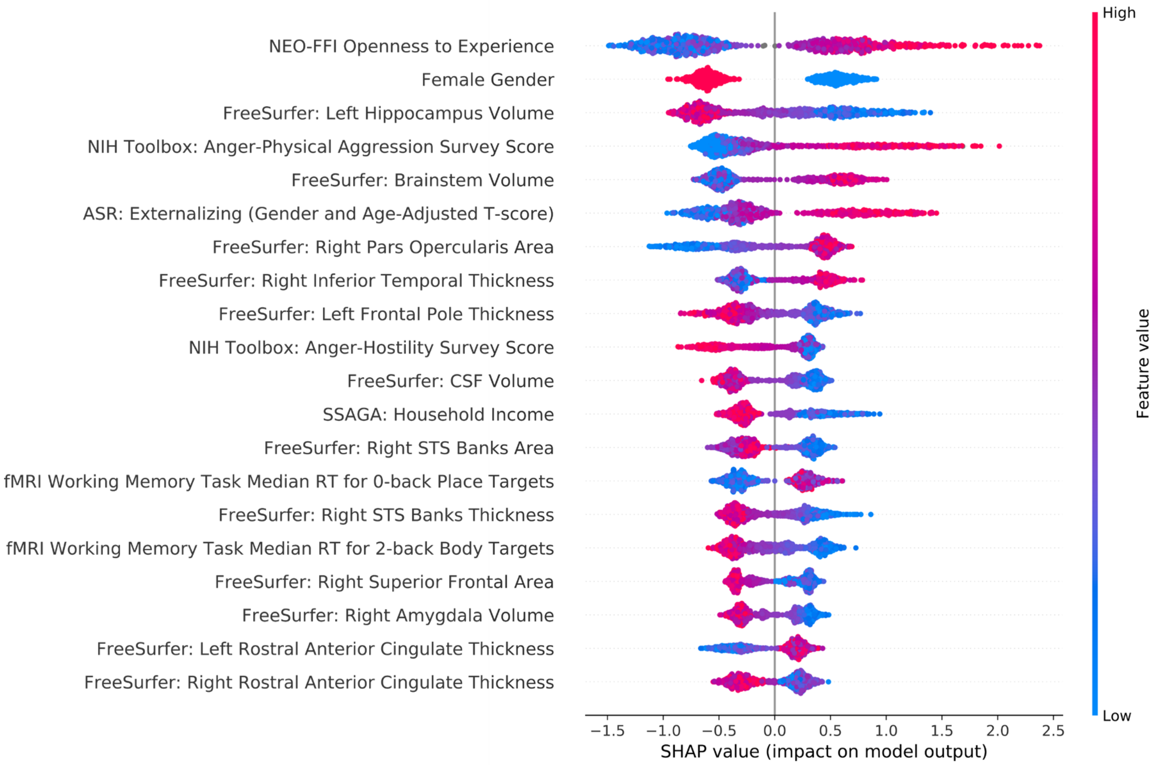

Supplementary Figure 3. *SHAP factor ranking for the phenotypic+Freesurfer model classifying 10+ lifetime cannabis uses. More positive SHAP values on the x-axis indicate that the observed factor pushed the classification closer towards cannabis use, whereas more negative SHAP values indicate that the factor pushed the classification away from cannabis use. Factors are ranked in order of greatest average SHAP value, which indicates the importance of the factor. Individual points represent the model output for each individual in the sample. The position of a dot on the x-axis represents the impact of the observed factor on the model output for the individual. The color of the individual dots represent the value of the observed measurements, with blue indicating lower and red higher values.*

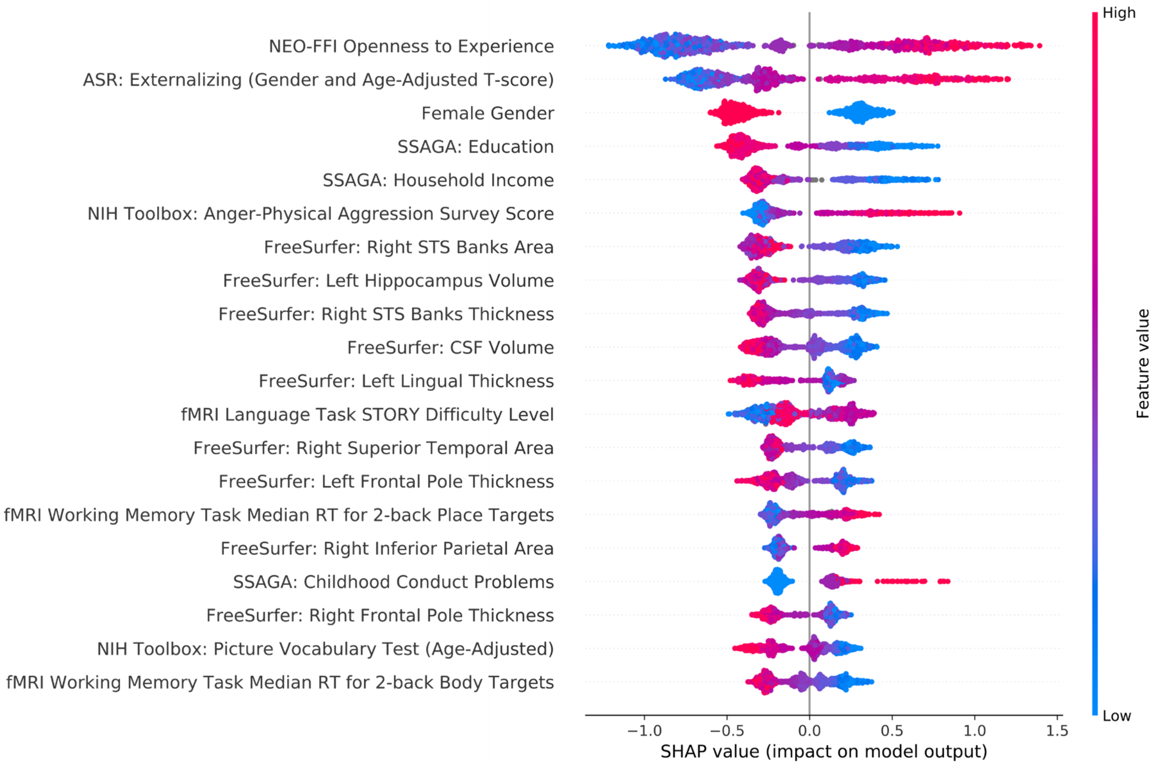

Supplementary Figure 4. *SHAP factor ranking for the phenotypic+Freesurfer model classifying 100+ lifetime cannabis uses. More positive SHAP values on the x-axis indicate that the observed factor pushed the classification closer towards cannabis use, whereas more negative SHAP values indicate that the factor pushed the classification away from cannabis use. Factors are ranked in order of greatest average SHAP value, which indicates the importance of the factor. Individual points represent the model output for each individual in the sample. The position of a dot on the x-axis represents the impact of the observed factor on the model output for the individual. The color of the individual dots represent the value of the observed measurements, with blue indicating lower and red higher values.*

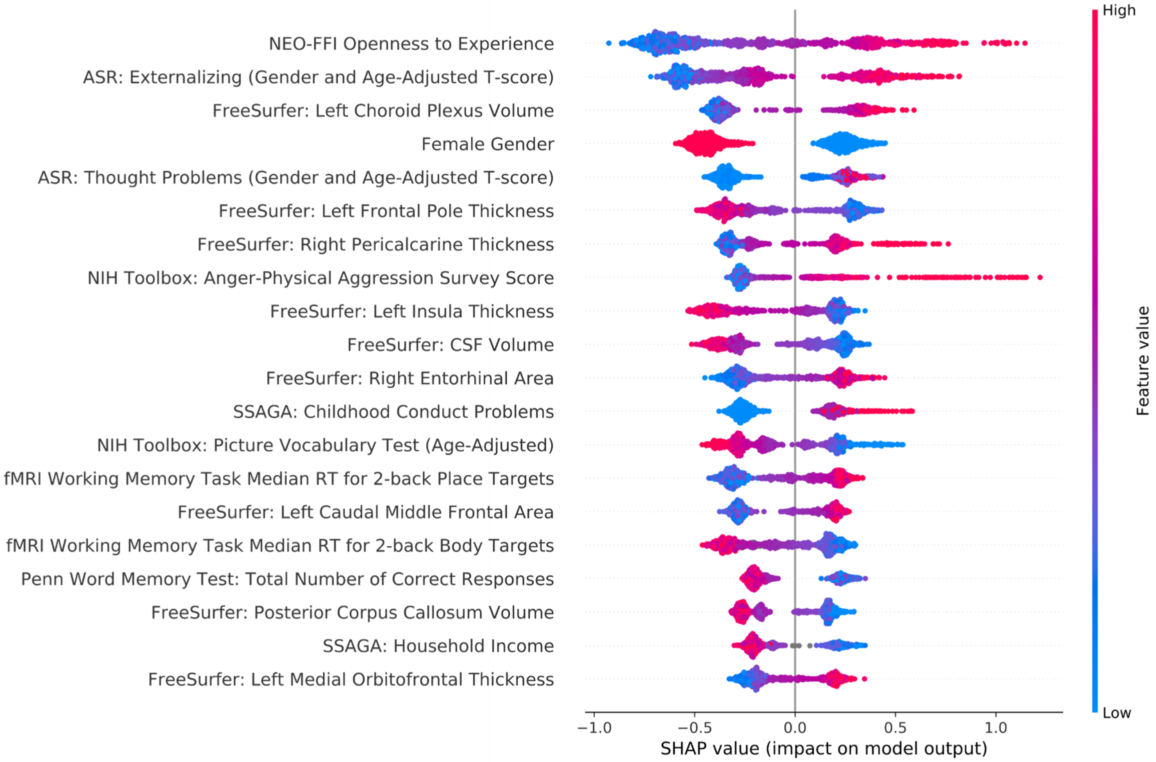

Supplementary Figure 5*. SHAP factor ranking for the phenotypic+Freesurfer model classifying 1000+ lifetime cannabis uses. More positive SHAP values on the x-axis indicate that the observed factor pushed the classification closer towards cannabis use, whereas more negative SHAP values indicate that the factor pushed the classification away from cannabis use. Factors are ranked in order of greatest average SHAP value, which indicates the importance of the factor. Individual points represent the model output for each individual in the sample. The position of a dot on the x-axis represents the impact of the observed factor on the model output for the individual. The color of the individual dots represent the value of the observed measurements, with blue indicating lower and red higher values.*

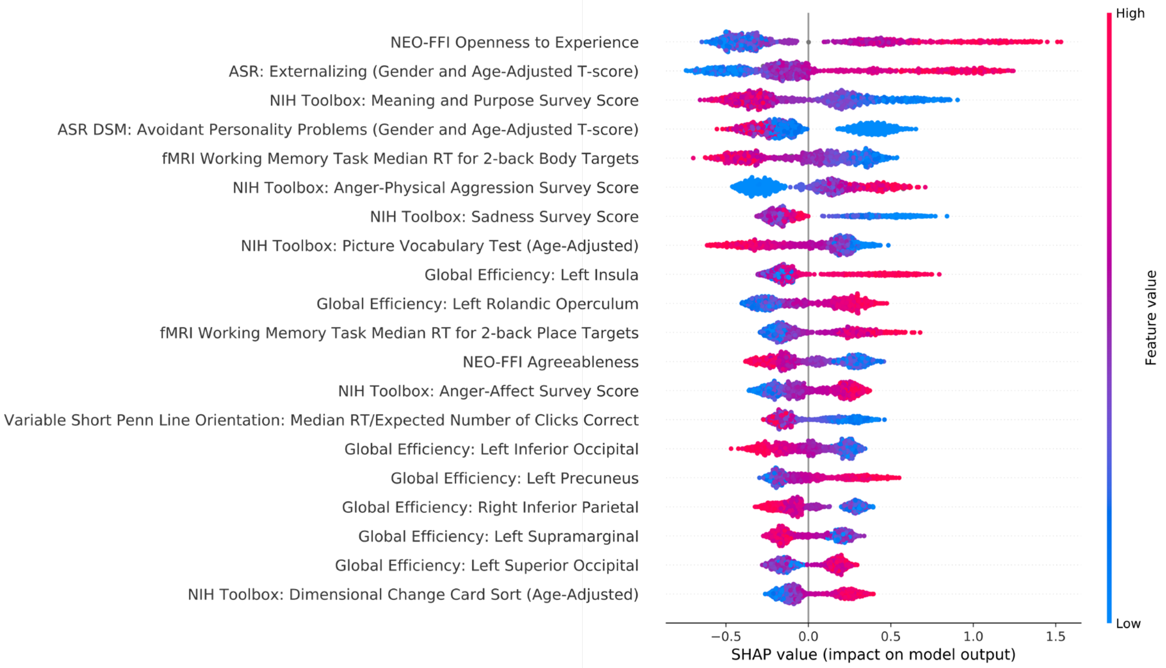

Supplementary Figure 6. *SHAP factor ranking for the phenotypic+Global Efficiency model classifying 1+ lifetime cannabis uses. More positive SHAP values on the x-axis indicate that the observed factor pushed the classification closer towards cannabis use, whereas more negative SHAP values indicate that the factor pushed the classification away from cannabis use. Factors are ranked in order of greatest average SHAP value, which indicates the importance of the factor. Individual points represent the model output for each individual in the sample. The position of a dot on the x-axis represents the impact of the observed factor on the model output for the individual. The color of the individual dots represent the value of the observed measurements, with blue indicating lower and red higher values.*

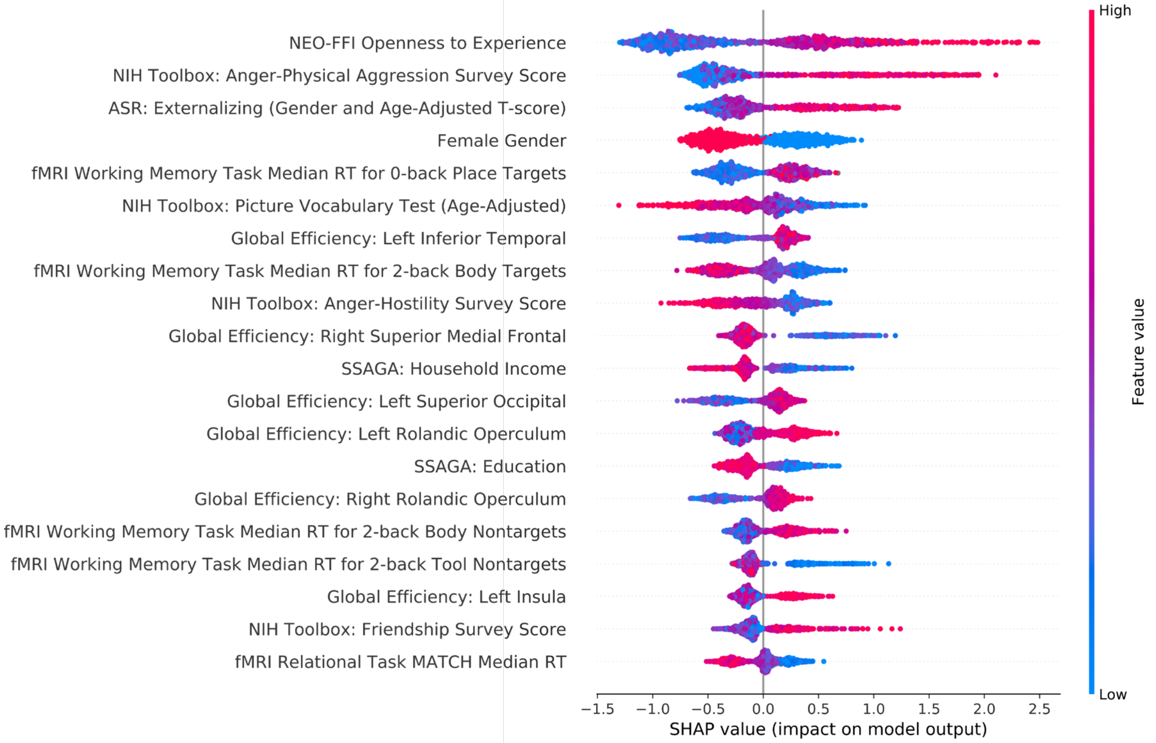

Supplementary Figure 7. *SHAP factor ranking for the phenotypic+Global Efficiency model classifying 10+ lifetime cannabis uses. More positive SHAP values on the x-axis indicate that the observed factor pushed the classification closer towards cannabis use, whereas more negative SHAP values indicate that the factor pushed the classification away from cannabis use. Factors are ranked in order of greatest average SHAP value, which indicates the importance of the factor. Individual points represent the model output for each individual in the sample. The position of a dot on the x-axis represents the impact of the observed factor on the model output for the individual. The color of the individual dots represent the value of the observed measurements, with blue indicating lower and red higher values.*

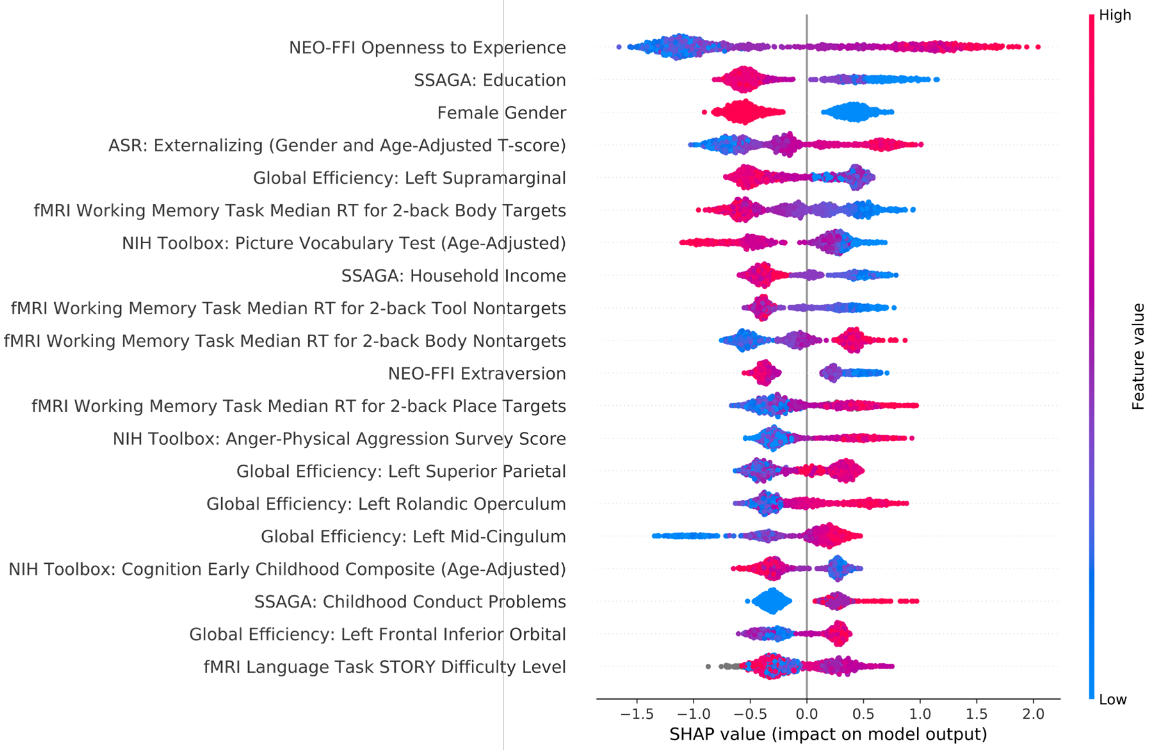

Supplementary Figure 8. *SHAP factor ranking for the phenotypic+Global Efficiency model classifying 100+ lifetime cannabis uses. More positive SHAP values on the x-axis indicate that the observed factor pushed the classification closer towards cannabis use, whereas more negative SHAP values indicate that the factor pushed the classification away from cannabis use. Factors are ranked in order of greatest average SHAP value, which indicates the importance of the factor. Individual points represent the model output for each individual in the sample. The position of a dot on the x-axis represents the impact of the observed factor on the model output for the individual. The color of the individual dots represent the value of the observed measurements, with blue indicating lower and red higher values.*

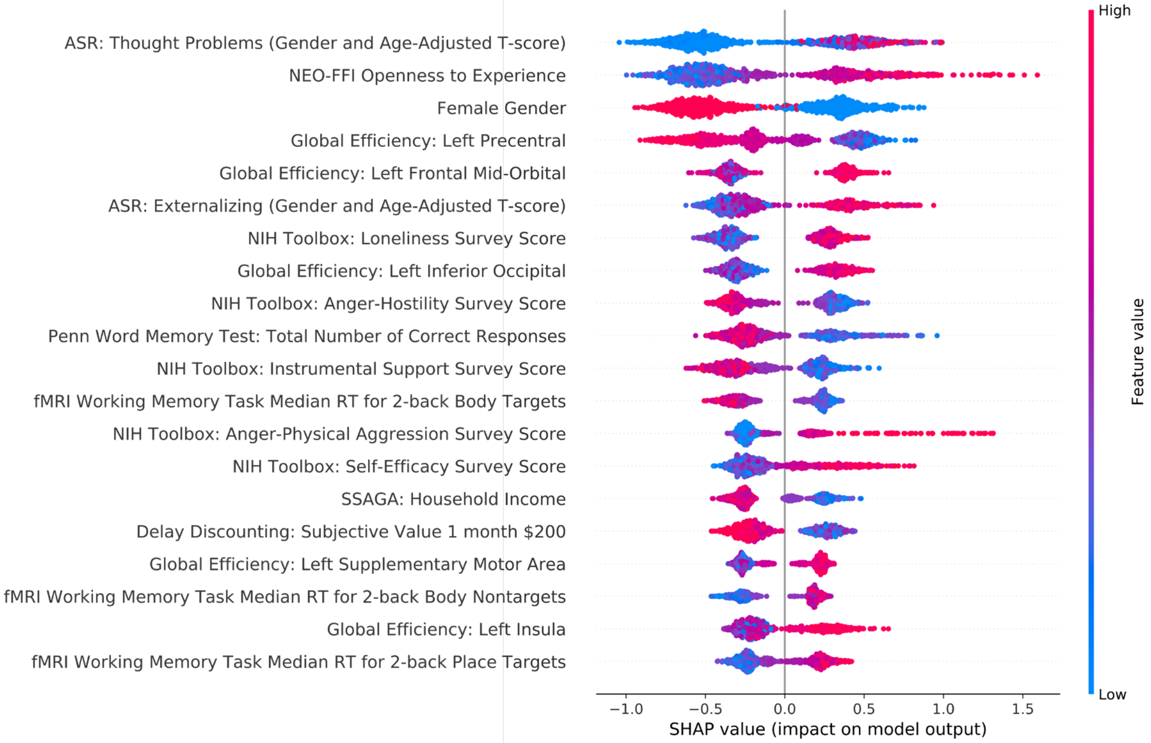

***Supplementary Figure 9**. SHAP factor ranking for the phenotypic+Global Efficiency model classifying 1000+ lifetime cannabis uses. More positive SHAP values on the x-axis indicate that the observed factor pushed the classification closer towards cannabis use, whereas more negative SHAP values indicate that the factor pushed the classification away from cannabis use. Factors are ranked in order of greatest average SHAP value, which indicates the importance of the factor. Individual points represent the model output for each individual in the sample. The position of a dot on the x-axis represents the impact of the observed factor on the model output for the individual. The color of the individual dots represent the value of the observed measurements, with blue indicating lower and red higher values.*
